# Characterization of the gut microbiome of wild *Peromyscus sonoriensis* in New Mexico, USA

**DOI:** 10.3389/frmbi.2026.1672092

**Published:** 2026-04-24

**Authors:** Andrew M. Skidmore, Samuel M. Goodfellow, Robert A. Nofchissey, Limin Jiang, Jonathan Dunnum, Joseph A. Cook, Yan Guo, Ivana Mali, Steven B. Bradfute

**Affiliations:** 1 Bradfute Lab, Center for Global Health, University of New Mexico Health Sciences Center, Albuquerque, NM, United States; 2 Department of Public Health and Sciences, University of Miami, Miami, FL, United States; 3 Museum of Southwestern Biology, Biology Department, University of New Mexico, Albuquerque, NM, United States; 4 College of Natural Resources, North Carolina State University, Raleigh, NC, United States

**Keywords:** model organisms, animal models, microbial ecology, field biology, non-human microbiomes, *Peromyscus sonoriensis*, western deer mouse

## Abstract

The microbiome is highly important to the physiologies of all multicellular organisms, particularly metazoans. However, the microbiomes of many wild animals remain understudied and poorly understood. *Peromyscus* mice are commonly used as models of adaptation, mental health, and human disease in biomedical research, and are also common in the environment across North America, frequently coming into close contact with humans. Additionally, *Peromyscus sonoriensis* are implicated as the primary reservoir for Sin Nombre hantavirus, a rare but severe disease of high morbidity and mortality in humans. Here we characterize the fecal microbiomes of 311 *Peromyscus sonoriensis*, the western deer mouse, collected from across New Mexico, USA, which will further their usefulness as models of disease and behavior as well as increase our understanding of their ecology. The animals used in this study are geographically diverse, collected from multiple ecoregions, and encompass mice of all ages and sexes. We sequenced the entire 16S rRNA gene in a PCR independent approach and characterized the microbiomes with Shannon entropy, Faith phylodiversity, and weighted UNIFRAC. We found that these mice have diverse microbiomes, with individuals varying in the presence and proportions of various identified bacteria. We analyzed the total population of mice according to age, sex, and trapping location, and found that trapping location was the only condition to significantly impact the microbiome. When the mice were subdivided by the location of collection, there were mild effects of age and sex. When comparing mice from archival museum storage, storage of samples in 95% ethanol resulted in significant alterations to the microbiome when compared to cryopreservation. Differential bacterial family presence was determined using ANCOMBC at the 0.05 significance threshold, and there were many differentially abundant families across all groups of mice. This data set can now be used as a reference for further research into the microbiomes of related *Peromyscus* species, enhance the use of *P. sonoriensis* as model laboratory animals, and as a source of novel research questions regarding the physiology of these rodents.

## 1 Introduction

To date, most research on microbiome detection and characterization has been performed in humans and in common laboratory animal models, such as the house mouse (*Mus musculus;* family Muridae). However, it has become increasingly clear that the focus of microbiome research on humans and laboratory model organisms is limiting our understanding of the relationships between commensal microbes and their hosts, as well as limiting our understanding of the true diversity of commensal microorganisms (Hird, 2017; Greyson-Gaito et al., 2020).

The microbiome is essential to the normal development of multicellular organisms and has profound and diverse effects on host physiology, behavior (Socała et al., 2021; Lu et al., 2022), immune system development (Davis et al., 2022; Yang et al., 2022; Lubin et al., 2023), and metabolic health (Qin et al., 2021; Michels et al., 2022; Fan et al., 2023), and can have strong effects on phenotypes in laboratory-reared model organisms (Ippolito et al., 2018; Mandal et al., 2023). It has been well established that rodents have significant alterations in their microbiomes after laboratory domestication (Bowerman et al., 2021). Multiple research groups are now refining approaches aimed at generating lab-reared *M. musculus* with microbiomes that more closely match those of their wild counterparts (Rosshart et al., 2019; Chen et al., 2023). Changes in the microbiome can alter the course of infectious disease in genetically identical animals, as well as the responses that organisms have to treatments and interventions (Nobs and Elinav, 2019; Rosshart et al., 2019). There is an extremely high rate of failure of preclinical drugs and disease interventions when moved to clinical trials in humans; less than 15% of preclinical drugs passed to phase I trials move to further development (Chi et al., 2022). This high attrition rate can be alleviated by colonization of laboratory-strain mice with wild-like microbiomes (Rosshart et al., 2019), indicating a possible avenue to improve models of infectious disease. Thus, it is imperative that we study the microbiomes of model organisms in their natural habitats so that we are able to mimic their commensal organisms in the laboratory.

In this paper we have characterized the microbiome of a model organism, the western deer mouse (*Peromyscus sonoriensis*; Family Cricetidae), formerly *Peromyscus maniculatus* (Bradley et al., 2019; Greenbaum et al., 2019). *Peromyscus* species are native to the Americas and highly diverse. Classification of *Peromyscus* is complex and there are many paraphyletic groups in the genus that may require elevation of currently recognized species to genus level and possible elevation of some groups to their own families (Greenbaum et al., 2019; Botten et al., 2000). *Peromyscus* mice are commonly used in studies of mental health, parenting, environmental adaptation, and disease (Botten et al., 2000; Wolmarans et al., 2018; Horvath et al., 2022; Arquilla et al., 2023; Bautista et al., 2024). Thus, the study of wild *Peromyscus* is necessary to better understand their use as animal models, as well as their basic biology in the wild.

Our work presented here is the first study of its kind examining the microbiomes of *Peromyscus sonoriensis*, which is common throughout the western United States and often syntopic with related species (Russell et al., 2022). These mice are widespread in North America, occupy a wide variety of ecological niches, and are highly tolerant of human ecological disturbance (Kalkvik et al., 2012; Witmer and Moulton, 2012). *P. sonoriensis* are also known to carry Sin Nombre hantavirus (Goodfellow et al., 2021), the most common cause of human hantavirus infections in North America (Whitmer et al., 2024). Their commonality in the environment, as well as their status as carriers of a severe zoonosis, makes these mice an important species for both field and laboratory studies. The goal of this study is to characterize the gut microbiome of wild *Peromyscus sonoriensis* and to determine major drivers of gut microbial diversity in these mice. This information can then be used in refinement of *Peromyscus* mice as model organisms, and compared to previous knowledge about the drivers of microbiome diversity in humans and laboratory mice.

Here we characterize the gut microbiomes of 311 wild *P. sonoriensis* mice collected over multiple years across New Mexico, USA, representing all *P. sonoriensis* captured by us at the time of DNA isolation, as well as compliment of archival samples selected for analysis. These mice inhabited a variety of biomes and areas with differing levels of human habitation and influence. We have leveraged the large sample size and geographical area to compare the gut microbial compositions of these mice with respect to several different variables, including sex, age, trapping location, and sample storage conditions. Based on previous literature, we hypothesized that changes in any of these variables would result in significant alterations to the gut microbiota (Maritan et al., ; Yatsunenko et al., 2012; Markle et al., 2013; Markle et al., 2013; Maurice et al., 2015; Trigunaite et al., 2015; Zheng et al., 2015; Leite et al., 2021; Marotz et al., 2021; Wu et al., 2021; Li et al., 2023; Worsley et al., 2024). We determined differences in alpha and beta diversity and what variables drove these changes while performing differential species analysis. Our work presented here provides a broad yet thorough overview of the bacterial and archaeal composition of the *P. sonoriensis* gut microbiome, which sets an important baseline for further analysis of these animals both in the wild and in the lab.

## 2 Materials and methods

### 2.1 Ethics statement

Animal field work was performed according to the guidance of the American Society of Mammalogists (Mills et al., 1995; Sikes et al., 2011). This work was approved by the University of New Mexico Institutional Animal Care and Use Committee (IACUC) protocol # 22–201287 and collection was carried out under New Mexico Department of Game and Fish permit: Authorization number 3300. Eastern New Mexico University samples were collected with the approval of New Mexico State University IACUC, protocol #2019–016 and New Mexico Department of Game and Fish permit: Authorization number 3621.

### 2.2 Animal collection protocol

*Peromyscus sonoriensis* were collected as described previously (Goodfellow et al., 2024; Goodfellow et al., 2025). Briefly, live Sherman traps (3 x 3.5 x 9in H.B. Sherman Co., Tallahassee, Florida) were placed and baited with a mixture of peanut butter and dried oats. Traps were set in the late evening and checked in the morning after each trap night. Animals were collected and samples processed the same day, immediately after euthanasia. Animals from the ENMU sites were euthanized using overdose of isoflurane followed by cervical dislocation (Banther-McConnell et al., 2024), all other animals were euthanized with chloroform vapors. Each trapping session consisted of one or two nights. Specimens are cataloged and archived in the Division of Mammals, Museum of Southwestern Biology.

When multiple trapping sessions were performed at a single site (McGaffey and Taos), all sessions were fully independent, and all individuals are fully independent data points other than originating from the same population.

### 2.3 Animal age classification and identification

The mice were divided into three age categories: adults, sub-adults and juveniles. Adults are fully grown and sexually mature; males have descended testicle, females have one of any of enlarged nipple, open vaginas, or large and developed uterine horns. Sub-adults are smaller than adults but are sexually mature, they may also maintain partial juvenile pelage color. Juveniles are not yet sexually mature and have distinct gray pelage color. *Peromyscus sonoriensis* age was based on total length (mm), weight, pelage color, and reproductive status. Age parameters were supported by the analysis of 490 P*. sonoriensis* collected throughout New Mexico. Age was based on size as follows: Adult (n = 212), average total length 160.4 mm (± 6.9 mm), average weight 20.2 g (± 4.1 g) Sub-adult (n = 165), average total length 145.7 mm (± 3.3 mm), average weight 17.4 g (± 3.5 g) and Juvenile (n = 113) average total length 130.4 mm (± 8.0 mm), average weight 12.4 g (± 2.9 g). Demographics of the collected mice were as follows: males 175, females 136, adults 215, sub-adults 42, juveniles 54. The use of pelage color as well as body size have been well established as methods for determine the relative age of multiple members of the *Peromyscus* genus (Colins, 1923; Dice, 1936; Dice and Bradley, 1942; Tabacaru et al., 2011).

Species were identified using morphological factors and measurements. This can be done with high accuracy even when closely related *Peromyscus* species exist in close sympatry with one another (Reed et al., 2004).

### 2.4 Feces collection

Fecal matter was collected from animals in our own field studies, as well as from samples that were archived in the Museum of Southwestern Biology. In our field studies loose feces was collected from the trap and after euthanasia if present. The colons of individual animals were removed and cryopreserved (-80 C) with any loose feces, if present.

Three methods were employed for feces collection from museum specimens: 1) large intestines of freshly collected animals were collected into individual sample tubes along with associated feces and loose feces if present. 2) Colons were removed from previously frozen whole animals. 3) Colons were removed from previously archived fluid preserved (95% EtOH) specimens.

Loose feces were prioritized but absent from most specimens. Colon-associated fecal matter was mechanically removed from the tissue. If samples lacked any collectible feces (a minority of samples), colon tissue was used (n = 19) from ENMU, Taos, Hopewell, El Malpais, and Pecos/Cowles locations. We targeted 50 mg of feces for DNA isolation.

### 2.5 Isolation of fecal bacterial DNA

All isolations were performed using the ZymoBIOMICS DNA miniprep kit (Zymo Research D4300) following manufacturer’s published protocol. Cells were disrupted using a BioSpec Mini-Beadbeater-16 or a Benchmark BeadBug 6. Isolated DNA was quantified using spectrometry analysis with either a NanoDrop (Thermofisher) or the Take-5 software with an Agilent plate reader.

### 2.6 Sequencing of the 16S rRNA gene

Isolated DNA was sent to the Genome Technology Access Center (GTAC) at Washington University St. Louis for sequencing and initial determination of bacterial species in their MVRSION protocol (Schriefer et al., 2018). After sequencing, sequences and initial species calling and analysis were provided. Species were identified to the either species or subspecies level depending on.

### 2.7 Bioinformatics analysis of microbiome data

All analysis was performed using the QIIME 2 software package (Bolyen et al., 2019). A frequency feature table was obtained directly from GTAC and was used to generate taxonomy plots and PCA plots (Lozupone and Knight, 2005; Lozupone et al., 2007; Hamady et al., 2010; McKinney, 2010; Halko et al., 2011; Lozupone et al., 2011; Legendre and Legendre, 2012; McDonald et al., 2012; Vázquez-Baeza et al., 2013; Vázquez-Baeza et al., 2017; Weiss et al., 2017; McDonald et al., 2018; Bolyen et al., 2019).

Analysis was focused on measurements of alpha diversity, beta diversity, and differential bacterial abundance. These analyses were performed in the Qiime2 software package.

For examination of sub-groups all animals were stratified using a positive selection strategy. For example, when analyzing adult mice, we selected only those mice that were tagged as adults and removed all other mice from the analysis.

We did not control for group size in our various analyses as each group analyzed had high absolute numbers of individuals regardless of relative group size. We also did not distinguish between museum samples and our own collections for identical storage conditions.

#### 2.7.1 Alpha diversity

Alpha diversity analysis was used to determine the average diversity of the microbiome of mice in different categories, both the Shannon entropy and Faith phylodiversity were used. Shannon is weighted to deemphasize rare occurrences, and Faith considers more related bacteria to be more similar in diversity calculation. Faith is only reported if it was significant while Shannon found no significant differences. Significance was calculated using the Kruskal-Wallis with multiple testing correction for pairwise analysis (Shannon, 1948; Kruskal and Wallis, 1952; Faith, 1992; McKinney, 2010; McDonald et al., 2012; Weiss et al., 2017; Bolyen et al., 2019).

#### 2.7.2 Beta diversity

Beta diversity analysis was used to determine the similarity in composition of the microbiomes of mice in different groups. Beta diversity was calculated using the weighted unique fraction metric (UNIFRAC). This measure is weighted to deemphasize rare occurrences and incorporates phylogenetic information and considers closely related bacteria more similar in diversity than distantly related bacteria. Significance was calculated using the PERMANOVA test with 999 permutations, with multiple testing correction for pairwise analysis (Anderson, 2001; Lozupone and Knight, 2005; Lozupone et al., 2007; Hamady et al., 2010; McKinney, 2010; Lozupone et al., 2011; McDonald et al., 2012; Weiss et al., 2017; McDonald et al., 2018; Bolyen et al., 2019).

#### 2.7.3 Differential abundance

Differential abundance was calculated using the method of analysis of microbiome composition with bias correction (ANCOMBC). Differential present bacteria were considered significant if they met the p = 0.05 significance threshold (McKinney, 2010; McDonald et al., 2012; Bolyen et al., 2019; Lin and Peddada, 2020).

#### 2.7.4 Multivariant statistical analysis and generalized mixed models

To account for potential interactions among multiple factors, we employed Generalized Linear Mixed Models (GLMMs), implemented via the lme4 and ANCOMBC R packages, to assess the influence of sample-, host-, and ecology-related variables on alpha diversity, beta diversity, and differential abundance. Body weight was used solely for species identification and age determination of rodents and was therefore not included as an independent variable in these analyses.

Alpha diversity models were as follows:

As GLMMS require a single value beta diversity matrices are incompatible with this analysis. To overcome this, we performed Principal Coordinates Analysis (PCoA) to transform the distance matrix into orthogonal axes (PCoA1–PCoA3), allowing for quantitative comparison of model performance across configurations.

Beta diversity models were as follows:

All R code can be found in the **Supplementary Materials**.

1. Sample-level model: shannon_entropy ~ ethanol + (1 | Museum_Sample).
2. Host-level model: shannon_entropy ~ Sex + Age + (1 | Museum_Sample).
3. Ecological-level model: shannon_entropy ~ Location + (1 | Museum_Sample).
4. Full model: shannon_entropy ~ ethanol + Sex + Age + Location + (1 | Museum_Sample).

1. Sample-level model: PCoA ~ ethanol + (1 | Museum_Sample).
2. Host-level model: PCoA ~ Sex + Age + (1 | Museum_Sample).
3. Ecological-level model: PCoA ~ Location + (1 | Museum_Sample).
4. Full model: PCoA ~ ethanol + Sex + Age + Location + (1 | Museum_Sample).

## 3 Results

### 3.1 Fecal microbiomes of *Peromyscus sonoriensis* are highly diverse

**Figure 1:**
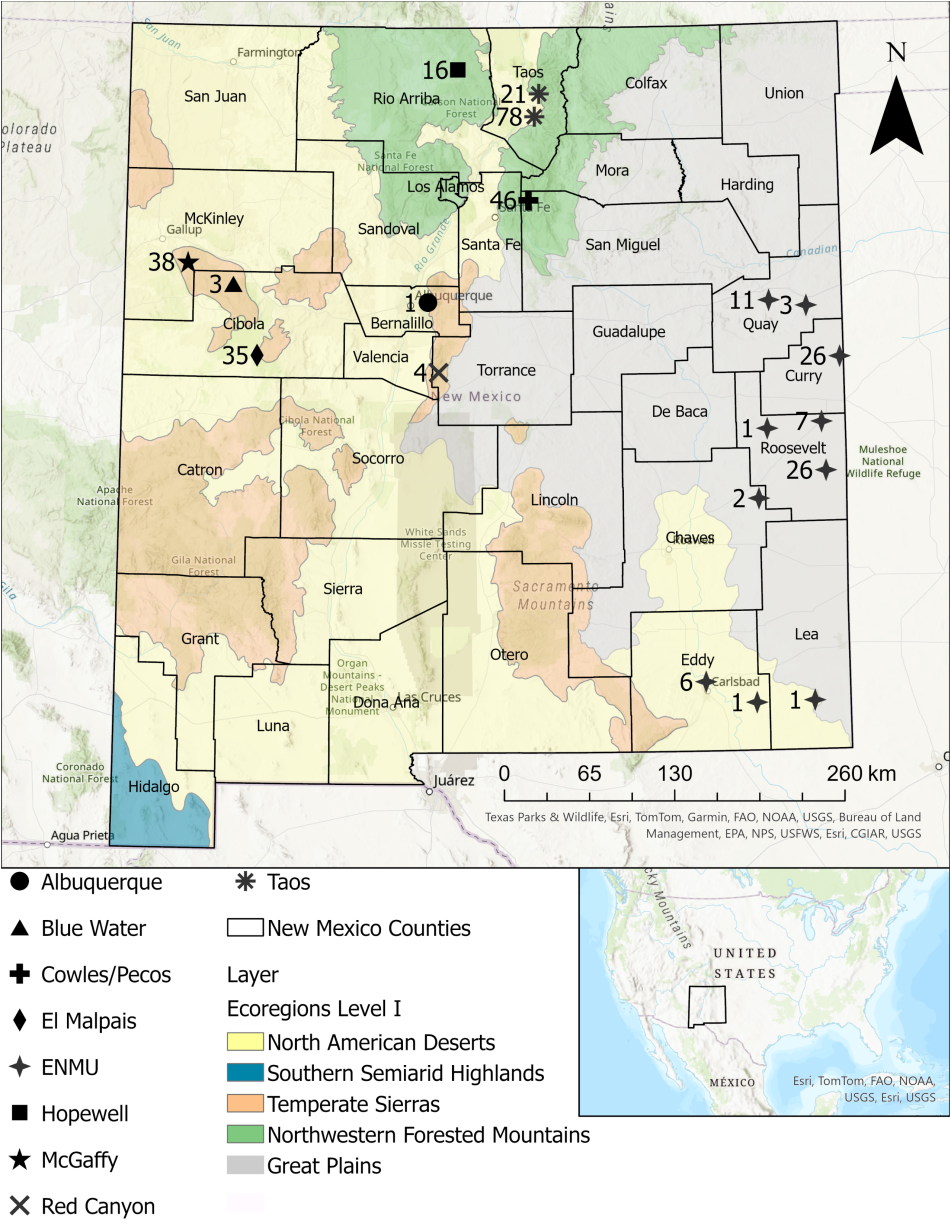
Trapping locations of *Peromyscus sonoriensis*. A map of New Mexico with labeled counties, trapping locations used in this study, and the level 1 ecoregions. United States map indicates the location of New Mexico within the country. Each location is labeled with the number of mice collected. Shapes correspond to the named locations as indicated in the key.

We collected feces from 311 live trapped *P. sonoriensis* at multiple sites across New Mexico (**Figure 1**). A summary of the demographics of the mice used in this study is found in **Table 1**.

**Table 1:** Demography of mice used in this study.

Demographic	Number of Mice
Male	175
Female	136
Adult	215
Sub-adult	42
Juvenile	54

**Figure 2:**
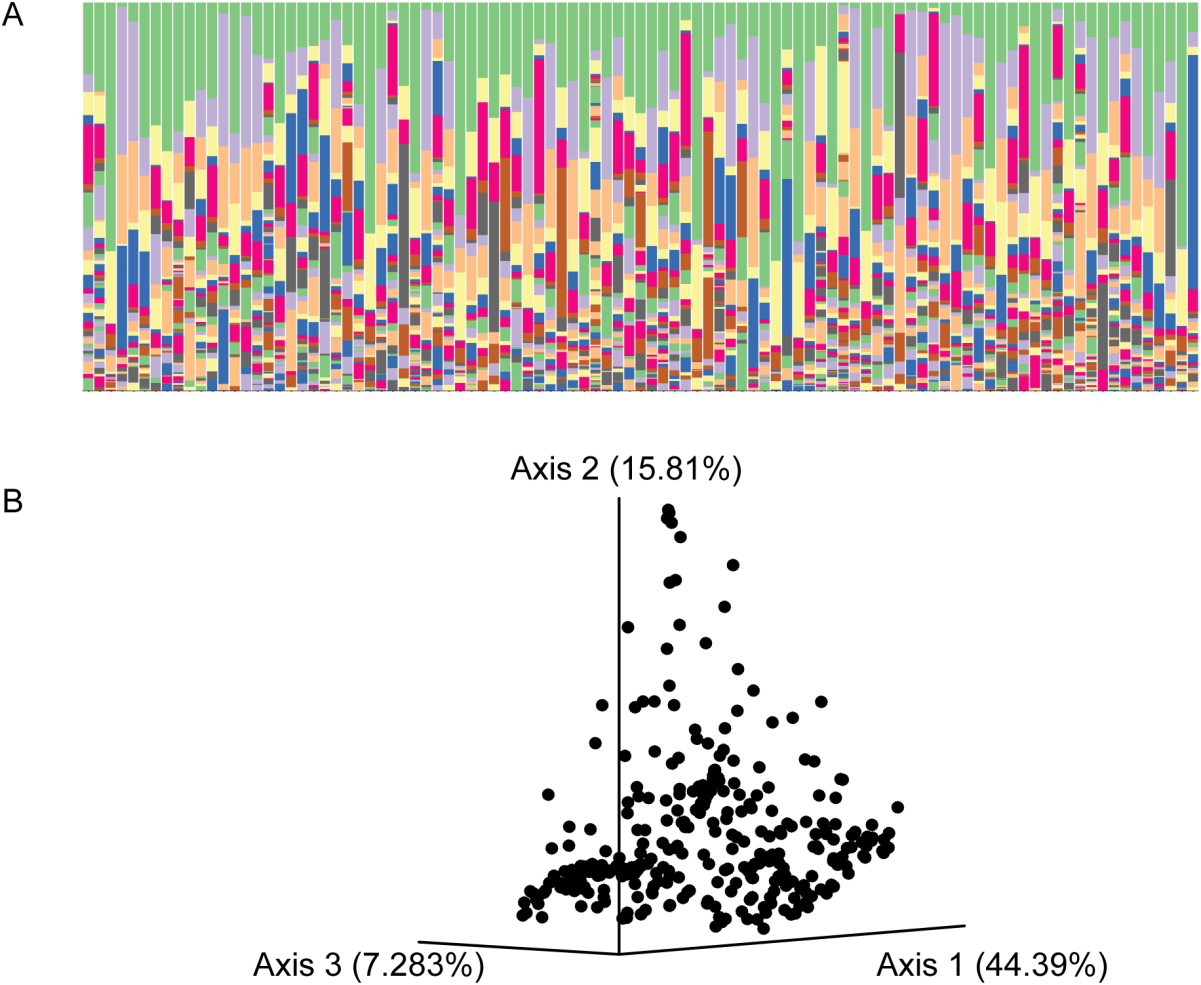
The gut microbiomes of wild *Peromyscus sonoriensis* from New Mexico are highly diverse. **(A)** Representative taxa bar plots of individual mice at the species level of bacterial identification. Ninety-nine individuals are shown, these were chosen at random from the sample. There is a large diversity in the species present as well as the different proportions of those species that are present. **(B)** A principal component analysis plot representing all mice included in this study. Total diversity equals 100% total displayed diversity equals 67.6%, 32.4% of the diversity is not explained by the first three components of the PCOA.

Initial analysis of these gut microbial communities indicated that individual mice have highly diverse and distinctive microbiomes (**Figure 2**). Each mouse has a unique complement of bacteria (**Figure 2A**) and diverse relative abundance of bacterial species. The individual mice also disperse relatively evenly after principal component analysis, with no apparent subpopulations (**Figure 2B**). These data are consistent with previous work indicating that individual microbiomes are highly variable even within individuals in the same population of a given species (Schloissnig et al., 2013; Weldon et al., 2015; Chen et al., 2021; Vandeputte et al., 2021).

**Figure 3:**
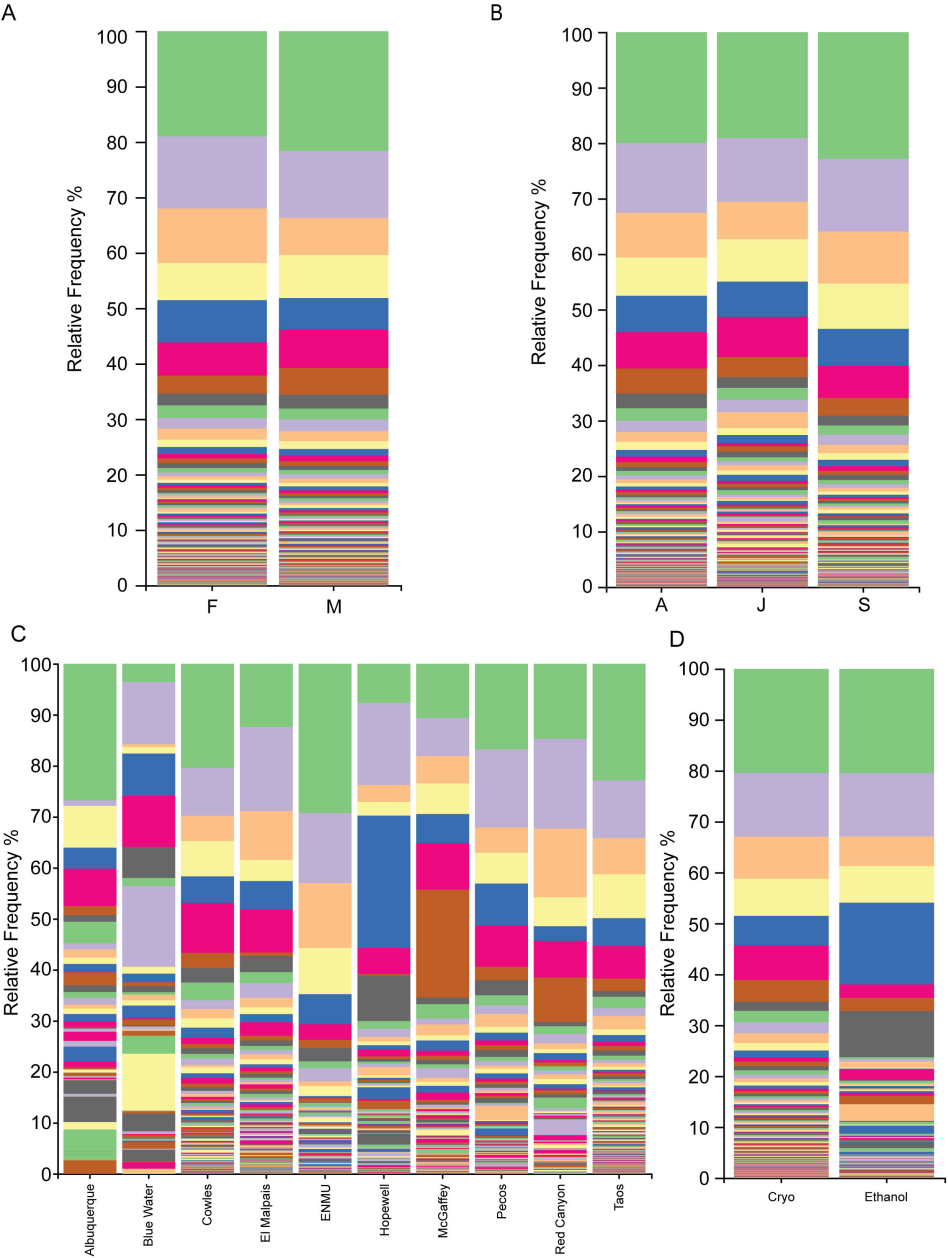
Grouped taxa plots of the microbiome of *P. sonoriensis* at the species level. **(A)** Taxa plots grouped by sex. There are minimal visual changes in the taxa plots in relation to sex. **(B)** Taxa plots grouped by age. There are minimal visual differences in the taxa plots when grouped by mouse age. **(C)** Taxa plot grouped by trapping location. There are obvious differences in the species of bacteria found in mice from different locations, both in presence and proportion. **(D)** Taxa plots grouped by storage condition. Samples that were stored in ethanol display obvious differences in their microbiomes compared to those that were cryopreserved.

When taxa bar plots were generated from grouped mice, some commonalities were apparent (**Figure 3**). Although mice of different sexes and ages had similar microbial compositions, (**Figures 3A, B**), mice from different geographical locations appear to vary in taxa makeups (**Figure 3C**). There are also obvious effects from storage of samples in ethanol compared to cryopreservation, as ethanol stored samples are from a variety geographic locations (**Figure 3D**).

### 3.2 Trapping location has a strong effect on the fecal microbiome of *P. sonoriensis*

Mice were collected from across New Mexico from the following different ecoregions: North American deserts, temperate sierras, and northwestern forested mountains. Named groupings of mice and number of animals in each can be found in **Table 2**.

**Table 2:** Number of mice from each named location.

Trapping Location	Number of mice
Albuquerque	1
Blue Water	3
Cowles	22
El Malpais	35
ENMU	69
Hopewell	16
McGaffey	38
Pecos	24
Red Canyon	4
Taos	99

**Figure 4:**
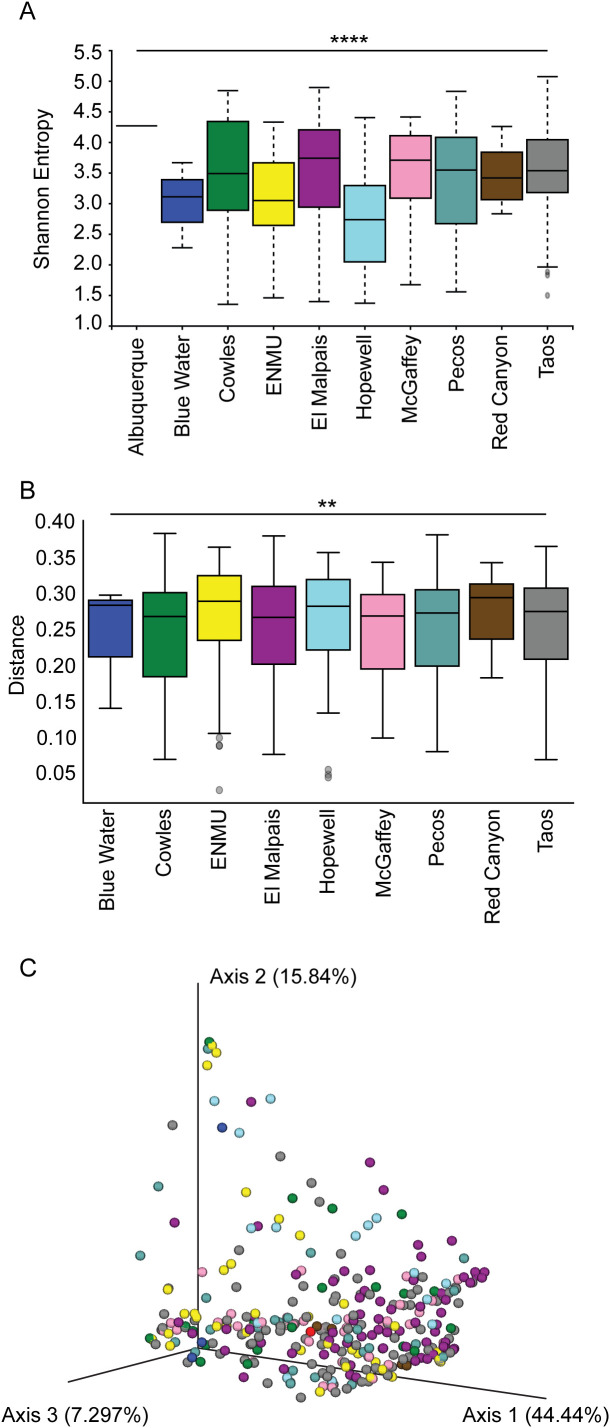
*P. sonoriensis* from different locations have distinct microbiomes. **(A)** Shannon Entropy of the different trapping locations. Different locations have significant alterations in their alpha diversities, p = 0.0004. **(B)** Beta diversity represented as the weighted UNIFRAC distance. Mice from different locations have significantly different bacterial compositions of their microbiomes as determined by PERMANOVA with 1000 permutations, p = 0.01. **(C)** PCA plot of all individual mice coded by location. Despite significant differences in their beta diversities, no single location populations are readily apparent. *P ≤ 0.05, **P ≤ 0.01, ***P ≤ 0.001, ****P ≤ 0.0001.

The strong, significant relationship between alpha diversity and trapping location (**Figure 4A**, overall Shannon p = 0.0004) is also reflected in the q values (multiple comparison corrected significance values) from pairwise location testing (**Table 3**). This difference appears largely driven by the ecoregion of a given trapping location, as can be seen in the differences in the alpha diversity between the ENMU animals and many of our other trap sites, q ≤ 0.05. The ENMU collection sites were located primarily in the dry Great Plains, with several animals from the North American Deserts ecoregion as well, whereas the other non-ENMU sites were predominantly in New Mexico’s Temperate Sierras and Northwestern Forested Mountains. This effect of ecoregions can also be seen in the difference between mice from Hopewell and Taos, q = 0.0195. Although close to each other, the Taos sites are located on the border between more arid desert and more mesic Forested Mountains (**Figure 1**), whereas Hopewell is located well within the Forested Mountains ecoregion.

**Table 3:** Location alpha and beta diversity pairwise comparisons.

Location 1	Location 2	q value alpha diversity	Significant alpha diversity difference	q value beta diversity	Significant beta diversity difference
Albuquerque	Blue Water	0.4493	No		
Albuquerque	Cowles	0.8440	No		
Albuquerque	ENMU	0.4407	No		
Albuquerque	El Malpais	0.6239	No		
Albuquerque	Hopewell	0.4493	No		
Albuquerque	McGaffey	0.4493	No		
Albuquerque	Pecos	0.5416	No		
Albuquerque	Red Canyon	0.4493	No		
Albuquerque	Taos	0.5416	No		
Blue Water	Cowles	0.6339	No	0.2930	No
Blue Water	ENMU	0.9214	No	0.0060	Yes
Blue Water	El Malpais	0.5416	No	0.3432	No
Blue Water	Hopewell	0.8102	No	0.0456	Yes
Blue Water	McGaffey	0.4493	No	0.0295	Yes
Blue Water	Pecos	0.6601	No	0.2310	No
Blue Water	Red Canyon	0.5416	No	0.0466	Yes
Blue Water	Taos	0.5110	No	0.0332	Yes
Cowles	ENMU	0.0853	No	0.0120	Yes
Cowles	El Malpais	0.9159	No	0.7856	No
Cowles	Hopewell	0.0853	No	0.0466	Yes
Cowles	McGaffey	0.8440	No	0.3330	No
Cowles	Pecos	0.6888	No	0.9330	No
Cowles	Red Canyon	0.8732	No	0.3432	No
Cowles	Taos	0.9159	No	0.2029	No
ENMU	El Malpais	0.0344	Yes	0.0060	Yes
ENMU	Hopewell	0.4493	No	0.0090	Yes
ENMU	McGaffey	0.0101	Yes	0.0060	Yes
ENMU	Pecos	0.3234	No	0.0060	Yes
ENMU	Red Canyon	0.5416	No	0.5513	No
ENMU	Taos	0.0031	Yes	0.0060	Yes
El Malpais	Hopewell	0.0432	Yes	0.0332	Yes
El Malpais	McGaffey	0.8708	No	0.1000	No
El Malpais	Pecos	0.8015	No	0.9237	No
El Malpais	Red Canyon	0.8708	No	0.3330	No
El Malpais	Taos	0.8440	No	0.0144	Yes
Hopewell	McGaffey	0.0277	Yes	0.0090	Yes
Hopewell	Pecos	0.2342	No	0.0334	Yes
Hopewell	Red Canyon	0.4493	No	0.1705	No
Hopewell	Taos	0.0195	Yes	0.0060	Yes
McGaffey	Pecos	0.8015	No	0.2310	No
McGaffey	Red Canyon	0.9114	No	0.3330	No
McGaffey	Taos	0.8708	No	0.1818	No
Pecos	Red Canyon	0.9159	No	0.3739	No
Pecos	Taos	0.8087	No	0.2029	No
Red Canyon	Taos	0.8708	No	0.7025	No

Black shading indicates these analyses were not completed.

We found a strong and significant relationship between location and beta diversity of the fecal microbiome (**Figures 4A, B**, overall weighted UNIFRAC p = 0.01), which is in contrast to the lack of separation of populations when visualized using principal component analysis (**Figure 4C**). The pairwise populations comparisons are similar to the alpha diversity results, with ecologically similar locations having similar beta diversities (**Table 3**).

### 3.3 There are no sex differences in the fecal microbiome of *P. sonoriensis* mice

Sex is an important driver of microbiome diversity in both humans and lab animals (Markle et al., 2013; Maurice et al., 2015; Kim, 2022). This is thought to be driven primarily by alterations in the immune systems due to the production of androgenic hormones which results in weaker inflammatory responses (Trigunaite et al., 2015; Kim, 2022). As this difference has been seen in multiple animal species, we hypothesized that the same differences would be seen in our samples as well.

**Figure 5:**
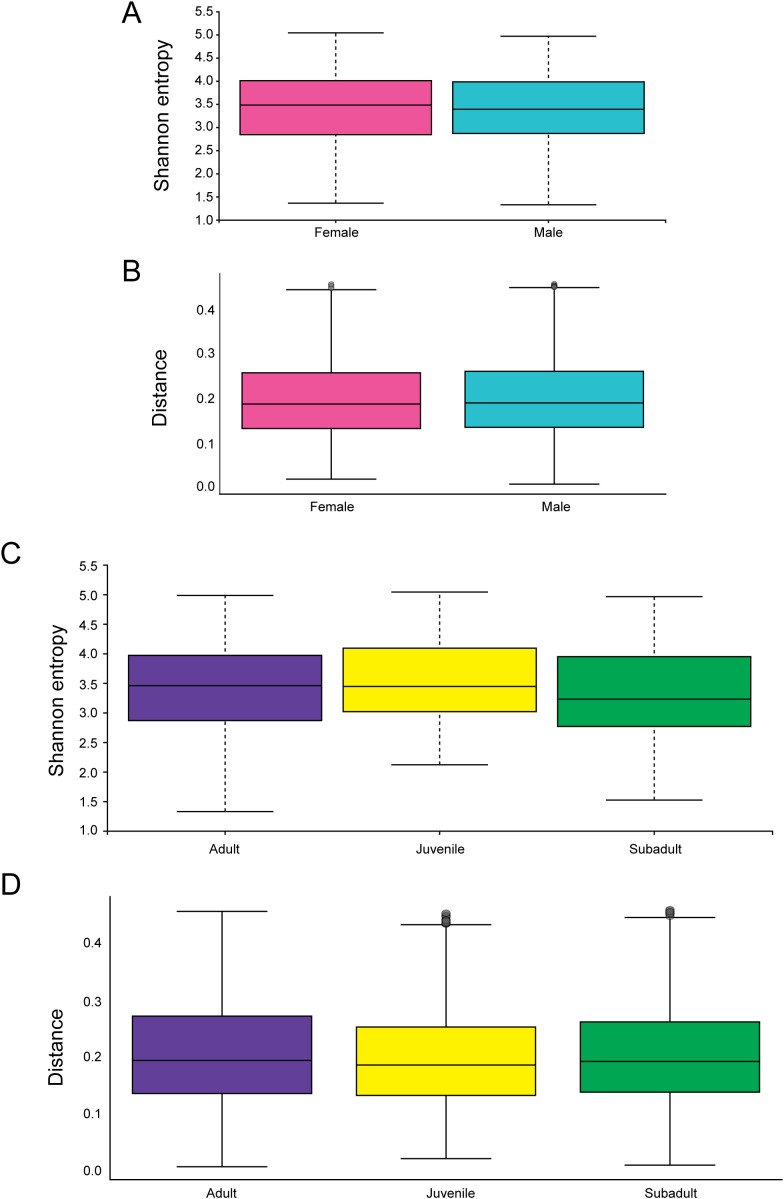
The microbiomes of *P. sonoriensis* mice show no significant differences in relation to sex or age. **(A)** Alpha diversities of male and female and female mice are not significantly different was measured by Shannon entropy or Faith phylodiversity. **(B)** Male and female mice have differences in their microbiome compositions as measure by weighted UNFIRAC. **(C)** There are no significant differences in the alpha diversity of mice across different age categories as measured by Shannon entropy or Faith phylodiversity. **(D)** Mice of different ages have no significant differences in their beta diversities as measured by weighted UNIFRAC.

Based on sex, alpha (**Figure 5A**) and beta (**Figure 5B**) diversities showed no difference (Shannon p = 0.46, weighted UNIFRAC p = 0.16). This absence of an effect of sex on the microbiomes of these mice is unusual (Kim, 2022). This lack of difference will require further study and characterization of how the estrogens and androgens of the *Peromyscus* species interreact with their immune systems, and potentially their microbiomes.

### 3.4 There is no significant difference in the microbiomes of mice across age classes

We categorize *P. sonoriensis* into three broad age classes, adults, juveniles, and sub-adults, as described in the methods. We hypothesized that each age of mouse would have distinct microbial commensals, because the microbiome has been shown to differ through life stages in both humans and lab mice (Yatsunenko et al., 2012; Leite et al., 2021; Wu et al., 2021; Worsley et al., 2024). This progression has generally followed the production of androgens and estrogens and begins around sexual maturity (Markle et al., 2013).

We compared these wild mice using alpha diversity (**Figure 5C**) and beta diversity (**Figure 5D**) and found no significant changes in the microbiomes of the mice when stratified by age (Shannon p = 0.411, weighted UNIFRAC p = 0.12).

### 3.5 Storage conditions of samples result in significant alterations to the microbiome

Ideally, isolation of bacterial DNA would occur immediately after sample collection. Barring this, cryopreservation with prevent growth of any commensal organisms. The majority of our samples were cryopreserved prior to DNA isolation.

However, 10% (Trigunaite et al., 2015) of our samples were preserved in 95% ethanol. Alcohol preservation is common, and it has been previously shown that microbial DNA can be retrieved from these samples (Greiman et al., 2018; Marotz et al., 2021; Li et al., 2023). It has also been shown that there are changes in the microbial populations that are found after ethanol preservation compared to cryopreservation. However, the nature of the changes in the bacterial community do not occur in a predictable manner (Marotz et al., 2021; Li et al., 2023).

**Figure 6:**
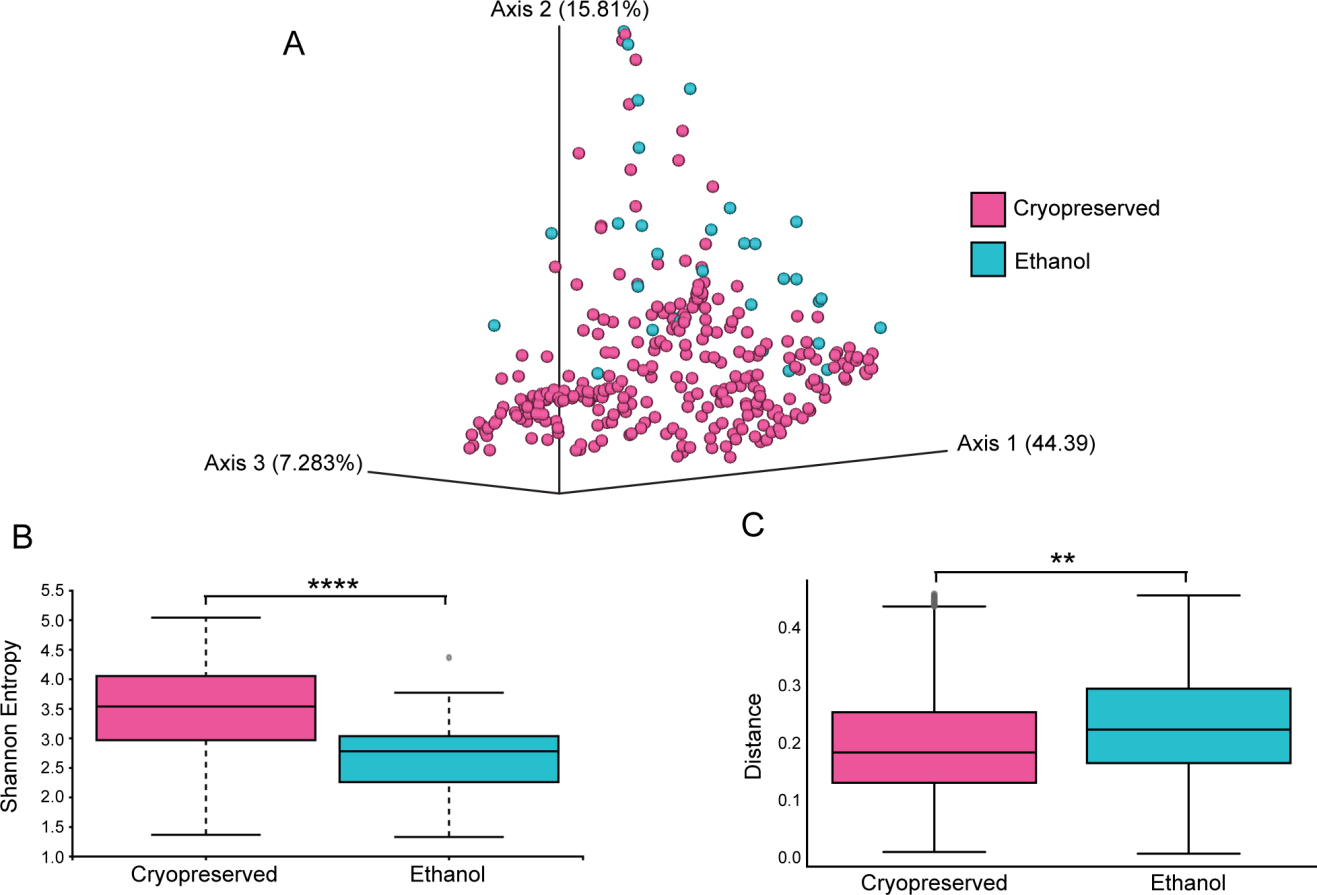
Storage conditions significantly alter the microbiomes of *P. sonoriensis* mice. **(A)** PCA plot indicating the storage condition for each sample. The samples that were stored in ethanol group away for the main body of the cryopreserved samples. **(B)** The samples that were stored in ethanol have significantly reduced alpha diversity as measured by Shannon entropy, p = 3.3 x10^-7^. **(C)** Samples that were stored in ethanol have significant compositional changes in their microbiomes as measured by weighted UNIFRAC, p = 0.01. *P ≤ 0.05, **P ≤ 0.01, ***P ≤ 0.001, ****P ≤ 0.0001.

Knowing this, we compared the alpha and beta diversities of the ethanol and cryopreserved samples (**Figure 6**). All locations with ethanol preserved samples also had cryopreserved samples from the same trapping sessions for comparison, and multiple locations had ethanol preserved samples. When the samples are plotted for PCA and identified by their storage conditions, there is grouping of the ethanol stored samples away from most of the cryopreserved samples (**Figure 6A**). When the alpha diversity is compared, the ethanol stored samples have significantly reduced diversities compared to those that were cryopreserved (**Figure 6B**, p = 3.3 x 10^-7^). Upon examining the beta diversity, there were significant compositional changes in the ethanol stored samples compared to the cryopreserved as well (**Figure 6C**, p = 0.01). These alterations together indicate that ethanol storage results in loss of diversity as well as significant compositional shifts in the bacterial community of the intestines. These alterations most likely occur at the level of bacterial shifts as DNA is highly stable in high concentrations of ethanol (Marquina et al., 2021).

#### 3.5.1 Storage-condition based alterations were maintained after sample categorization

We then parsed our samples based on their biological characteristics of sex and age (**Table 1**). The majority were male (56%). We also captured primarily adult mice (63%), followed by subadults (19%), and juveniles (18%).

**Figure 7:**
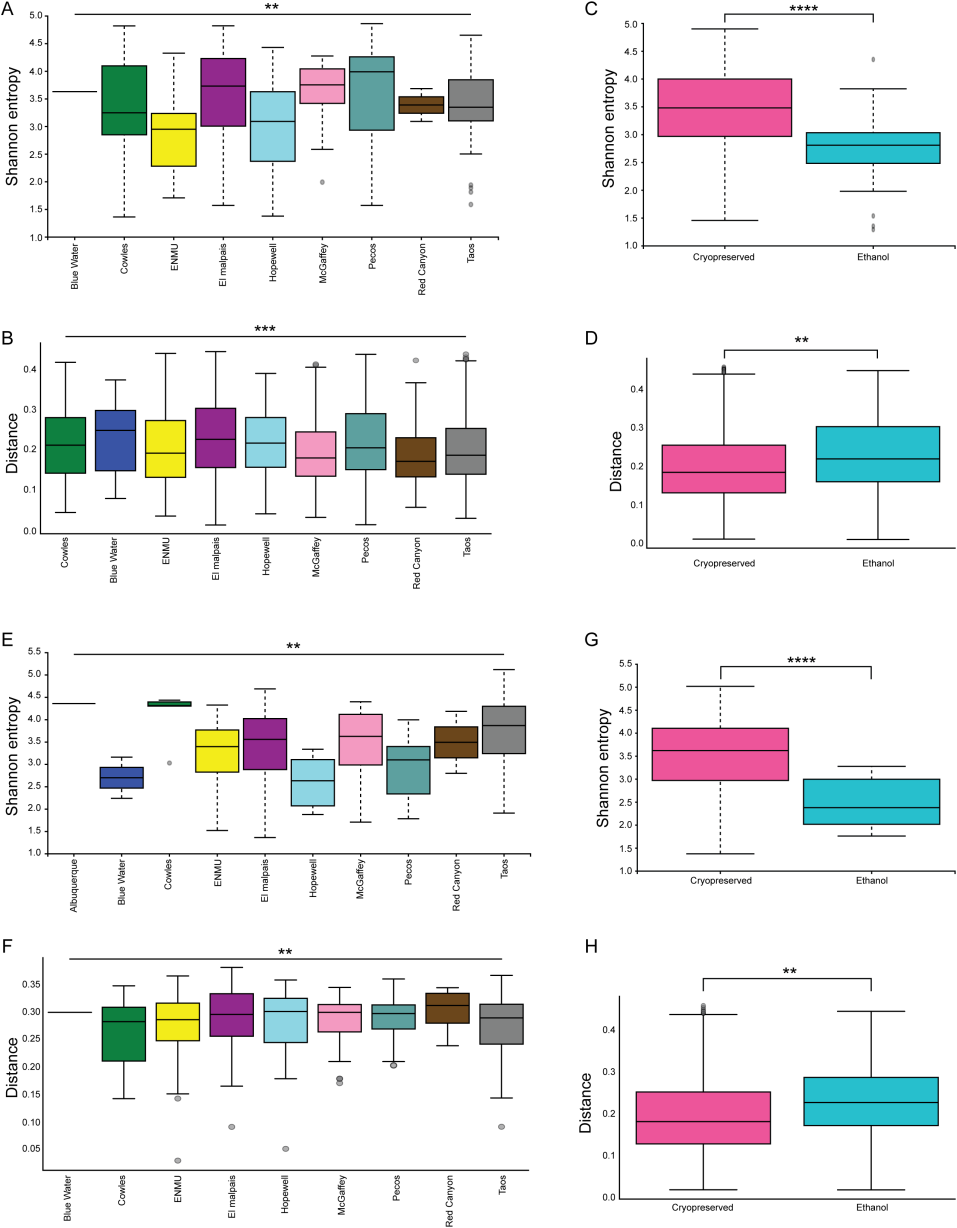
Division of *P. sonoriensis* mice into male and female does not reveal novel differences in their microbiomes. **(A)** Male mice from different trap sites have significantly different alpha diversities as measured by Shannon entropy, p = 0.005. **(B)** Male mice from different locations have significantly different beta diversities as measured by weighted UNIFRAC, p = 0.001. **(C)** Male mice stored ethanol have significantly reduced alpha diversity compared to cryopreserved, p = 2x10^-5^. **(D)** Male mice stored in ethanol have significantly different beta diversity compared to those that were cryopreserved, p = 0.01. **(E)** Female mice from different trapping locations have significantly different alpha diversities, p = 0.002. **(F)** Female mice from different locations have significantly different microbial compositions, p = 0.002. **(G)** Female mice stored in ethanol have significantly reduced alpha diversity compared to those that were cryopreserved, p = 0.00001. **(H)** Female mice stored in ethanol have significantly different beta diversity compared to those that were cryopreserved, p = 0.003. *P ≤ 0.05, **P ≤ 0.01, ***P ≤ 0.001, ****P ≤ 0.0001.

Male and female mice were analyzed separately (**Figure 7**). However, this division only resulted in the same variables being significant as were seen in the overall population. Male mice had significant differences in both alpha and beta diversity for location (**Figures 7A, B**, Shannon p = 0.005, weighted UNIFRAC p = 0.001). Male mice also had significant differences between ethanol fixation and cryopreservation (**Figures 7C, D**, Shannon p = 2 x 10^-5^, weighted UNIFRAC p = 0.01). Age of the male mice was not a significant factor in microbiome diversity.

Similar results were found for the female mice. Only location and storage conditions were found to be significant drivers of either the alpha or beta diversity of the microbial community (Location significance, **Figures 7E, F**, Shannon p = 0.002, weighted UNIFRAC p = 0.002; storage conditions, **Figures 7G, H**, Shannon p = 0.00001, weighted UNIFRAC p = 0.003).

**Figure 8:**
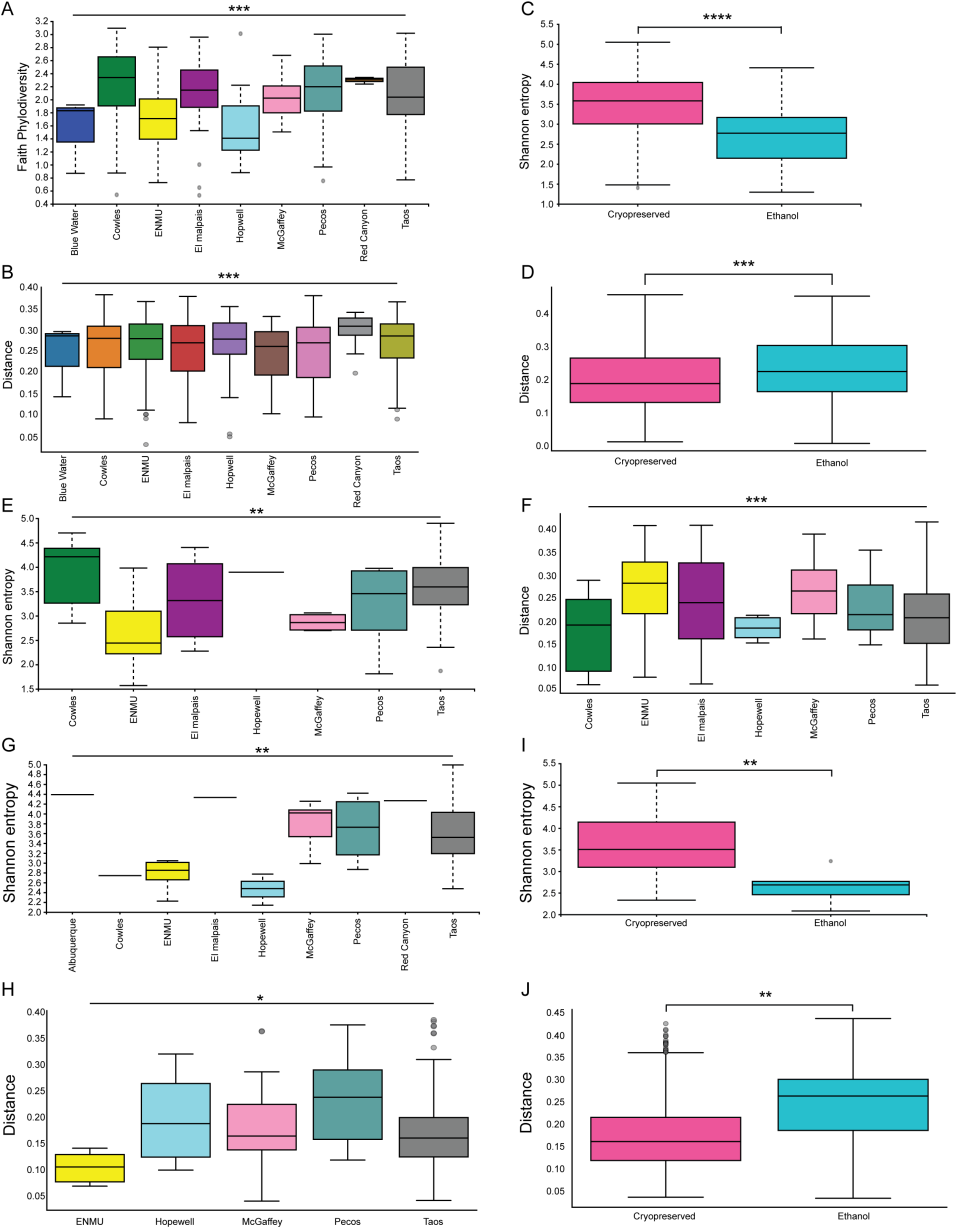
Division of *P. sonoriensis* mice into age categories does not find novel differences in their microbiomes. **(A)** Adult mice have significantly different alpha diversities as measured by Faith phylodiversity but not Shannon entropy, p = 0.0002. **(B)** Adult mice have significantly different beta diversities in different locations, p = 0.001. **(C)** Adult mice stored in ethanol have significantly lower alpha diversity, p = 2x10^-6^. **(D)** Adult mice stored in ethanol have significantly altered compositions of their microbiomes, p = 0.001. **(E)** Sub-adult mice from different locations have significantly different alpha diversities, Shannon entropy p = 0.01. **(F)** Sub-adult mice from different locations have significantly different beta diversities, p = 0.001. **(G)** Juvenile mice from different locations have significantly different alpha diversities, Shannon entropy p = 0.008. **(H)** Juvenile mice from different locations have significantly different beta diversities, p = 0.02. **(I)** Juvenile mice stored in ethanol had significantly reduced alpha diversity compared to those that were cryopreserved, p = 0.006. **(J)** Juvenile mice stored in ethanol have significantly altered beta diversity compared to those that were cryopreserved, p = 0.003. *P ≤ 0.05, **P ≤ 0.01, ***P ≤ 0.001, ****P ≤ 0.0001.

We next examined each age category of mice individually: adults, sub-adults, and juveniles (**Figure 8**). Each of these groups had the same variables investigated as the total population of mice. When the adult mice were grouped separately (**Figures 8A–D**), the storage conditions and location were once again found to be significant drivers of microbiome changes (location: Faith p = 0.0002, weighted, UNIFRAC p = 0.001; storage conditions: Shannon p = 2 x 10^-6^, weighted UNIFRAC p = 0.001).

Juveniles had similar results to the adults, with location and storage conditions resulting in differences (**Figures 8G–J**, location: Shannon p = 0.008, weighted UNIFRAC p = 0.02; storage conditions: Shannon p = 0.006, weighted UNIFRAC p = 0.003).

Sub-adult mice had the fewest differences. Sub-adult mice had distinctly uneven sampling with some locations having many, and some locations having few, if any, sub-adult mice collected. Sub-adult mice only had alterations in response to the location of capture, in both measures of alpha and beta diversity (**Figures 8E, F**, Shannon p = 0.01, weighted UNIFRAC p = 0.001).

### 3.6 Additional differences are observable after division of the mice by trapping location

Based on the previous analysis of the total mouse population as well as the mice grouped by biological variables, we found that the trapping location is a highly significant driver of differences in the microbiome as measured by both alpha and beta diversity. It is the only variable that was examined that resulted in significant alterations of the microbiome in every group of mice.

The consistency and strength of location effects led us to hypothesize that it could be masking the effects of weaker drivers of microbial diversity. To test this, we separated the mice by trapping location, then analyzed the different biological variables. Due to uneven trapping coverage at different locations, we were not able to subdivide to every location and instead focused on those with the largest number of *P. sonoriensis* collected. Specimens from Pecos and Cowles were also combined into a single named location as these locations are less than two kilometers apart from another and were trapped at the same time. ENMU was also grouped into a single location for analysis, as it largely encompasses a single ecoregion, and many discreet sites had too few animals to be examined separately.

However, our analysis of the individual locations resulted in minimal changes to the significant variables. Those grouped locations with significant differences had similar results at the whole population, with some locations having additional differences between animals of different ages and/or sexes. This was surprising to us, and it indicates that the microbiomes of *P. sonoriensis* are more similar within locations than we expected. This also further supports location as the strongest driver of microbial colonization in wild *P. sonoriensis*.

**Figure 9:**
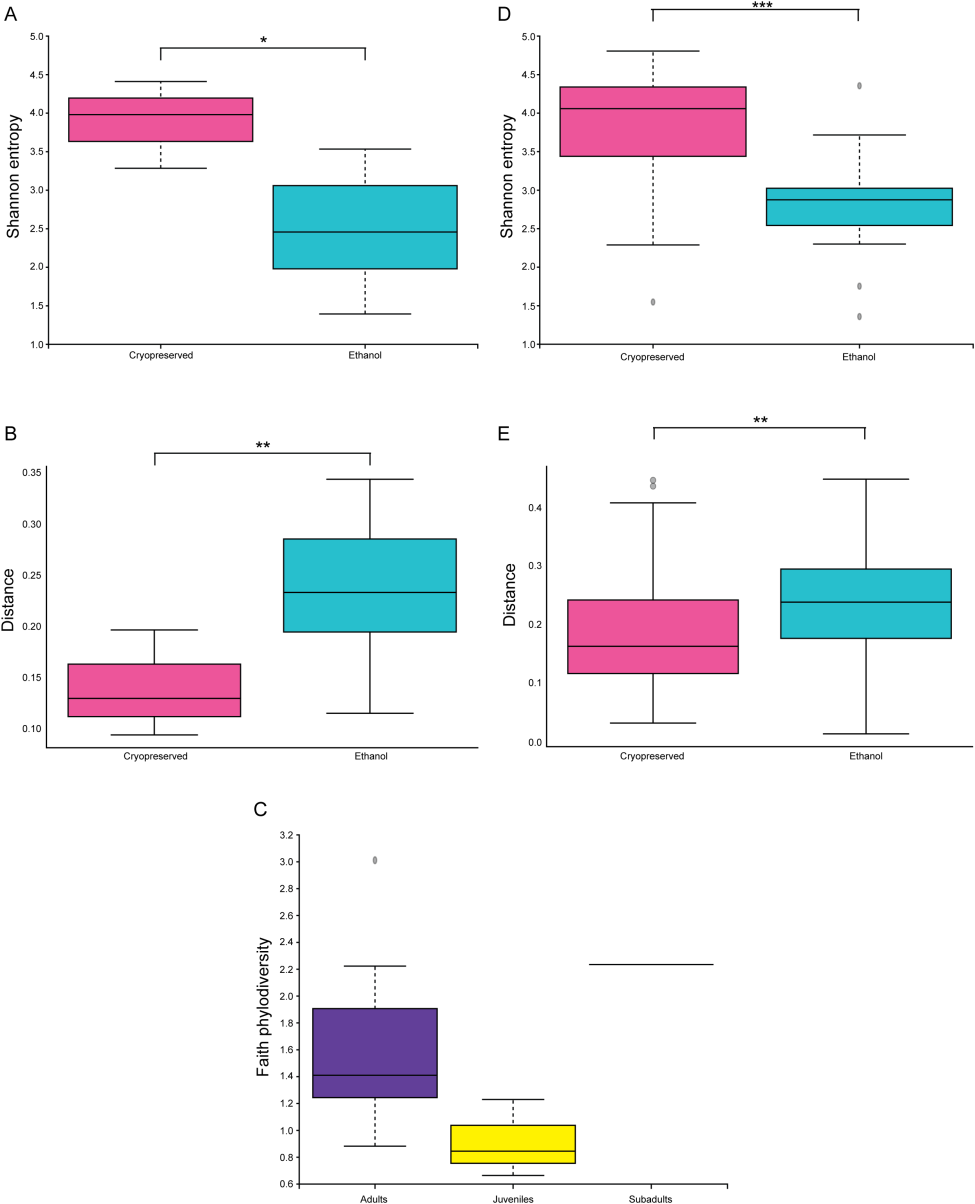
*P. sonoriensis* from Hopewell and Pecos/Cowels show minimal within-location differences. **(A)** Animals from Hopewell that were stored in ethanol have significantly reduced alpha diversity after storage in ethanol compared to cryopreservation, Shannon p = 0.02. **(B)** Animals from Hopewell that were stored in ethanol have significantly altered beta diversity compared to cryopreservation, p = 0.009. **(C)** Animals from Hopewell have significantly different alpha diversities in different age categories, Faith p = 0.04. **(D)** Animals from Pecos/Cowels have significantly reduced alpha diversity after storage in ethanol compared to cryopreservation, Shannon p = 8x10^-5^. **(E)** Animals from Pecos/Cowels have significant differences in their beta diversity after storage in ethanol compared to cryopreservation, p = 0.002. *P ≤ 0.05, **P ≤ 0.01, ***P ≤ 0.001, ****P ≤ 0.0001.

Hopewell and Pecos/Cowles had few differences apparent after subdivision (**Figure 9**). Hopewell only had differences in storage conditions (**Figures 9A, B**, Shannon p = 0.02, weighted UNIFRAC p = 0.009), and in age (**Figure 8C**, Faith phylodiversity p = 0.04). Pecos/Cowles only displayed alterations due to storage conditions (**Figures 8D, E**, Shannon p = 8x10^-5^, weighted UNIFRAC p = 0.002).

#### 3.6.1 Trapping year and season impact the microbiome of mice from a given location

**Figure 10:**
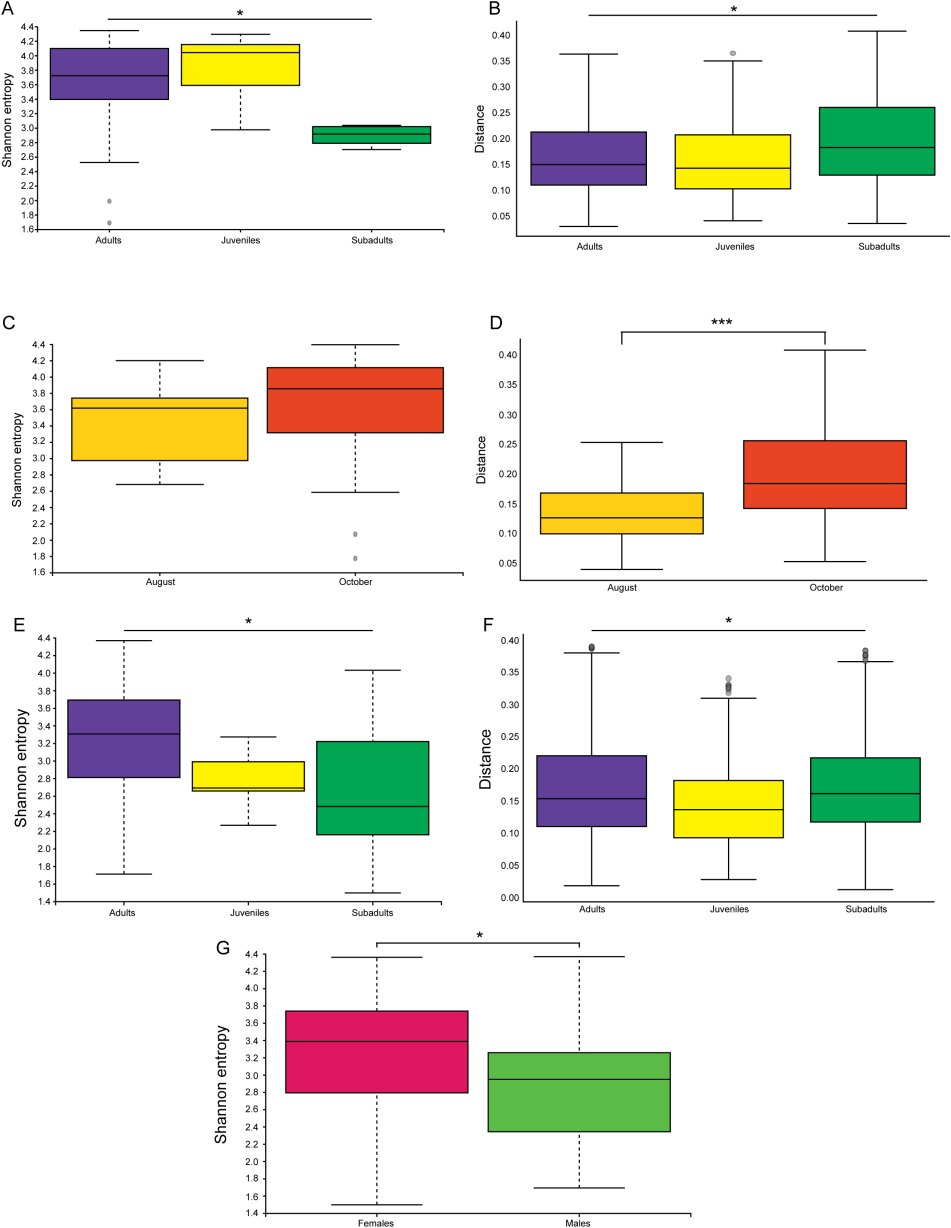
*P. sonoriensis* from McGaffey and ENMU have within-group differences in the microbiomes of mice of different ages, seasons, and sexes. **(A)** Mice from McGaffey have significantly different alpha diversities in different age groups, Shannon p = 0.05. **(B)** Mice from McGaffey have significant differences in their beta diversities in different age categories, p = 0.04. **(C)** Month of capture had no effect on the alpha diversity of mice from McGaffey. **(D)** Mice from McGaffey had significantly different beta diversities depending on month of capture, p = 0.001. **(E)** Mice from ENMU had significantly different alpha diversities depending on their age categorization, p = 0.02. **(F)** Mice from ENMU in different age categories had significantly different beta diversities, p = 0.03. **(G)** Male mice from ENMU had significantly reduced alpha diversities compared to female mice, p = 0.03. *P ≤ 0.05, **P ≤ 0.01, ***P ≤ 0.001, ****P ≤ 0.0001.

McGaffey and Taos are the only two locations where there were repeated trapping sessions performed independently at different times. In McGaffey we trapped in two consecutive years, but at different times of year, and it has been previously seen that season of the year can influence the microbiome (Maurice et al., 2015). Our first collection was performed in August of 2022 and the second in late October of 2023. When we examined the mice from McGaffey separately, we found that there were significant differences in the microbiome due to age and between the two trapping sessions (**Figure 10**), there are no ethanol stored samples from McGaffey. Age was a significant factor in both alpha and beta diversity (**Figures 10A, B**, Shannon p = 0.05, weighted UNIFRAC p = 0.04). The trapping session was also significant with relation to beta diversity but not alpha diversity (**Figures 10C, D**, weighted UNIFRAC p = 0.001).

The collective ENMU locations have the second largest number of mice. These collections cover two ecoregions, the dry Great Plains, where a majority of the mice were collected, and North American Deserts. We chose to combine these mice for analysis as several of the individual trapping sites had too few animals to analyze independently. The location analysis of the total population shows this as a valid grouping when comparing the alpha and beta diversities (**Figure 2**). When these samples were analyzed as a group there are significant changes in the microbiome related to age (**Figures 10E, F**) and sex (**Figure 10G**); there are no ethanol stored samples from ENMU (age: Shannon p = 0.02, weighted UNIFRAC = 0.03; sex: Shannon p = 0.03).

**Figure 11:**
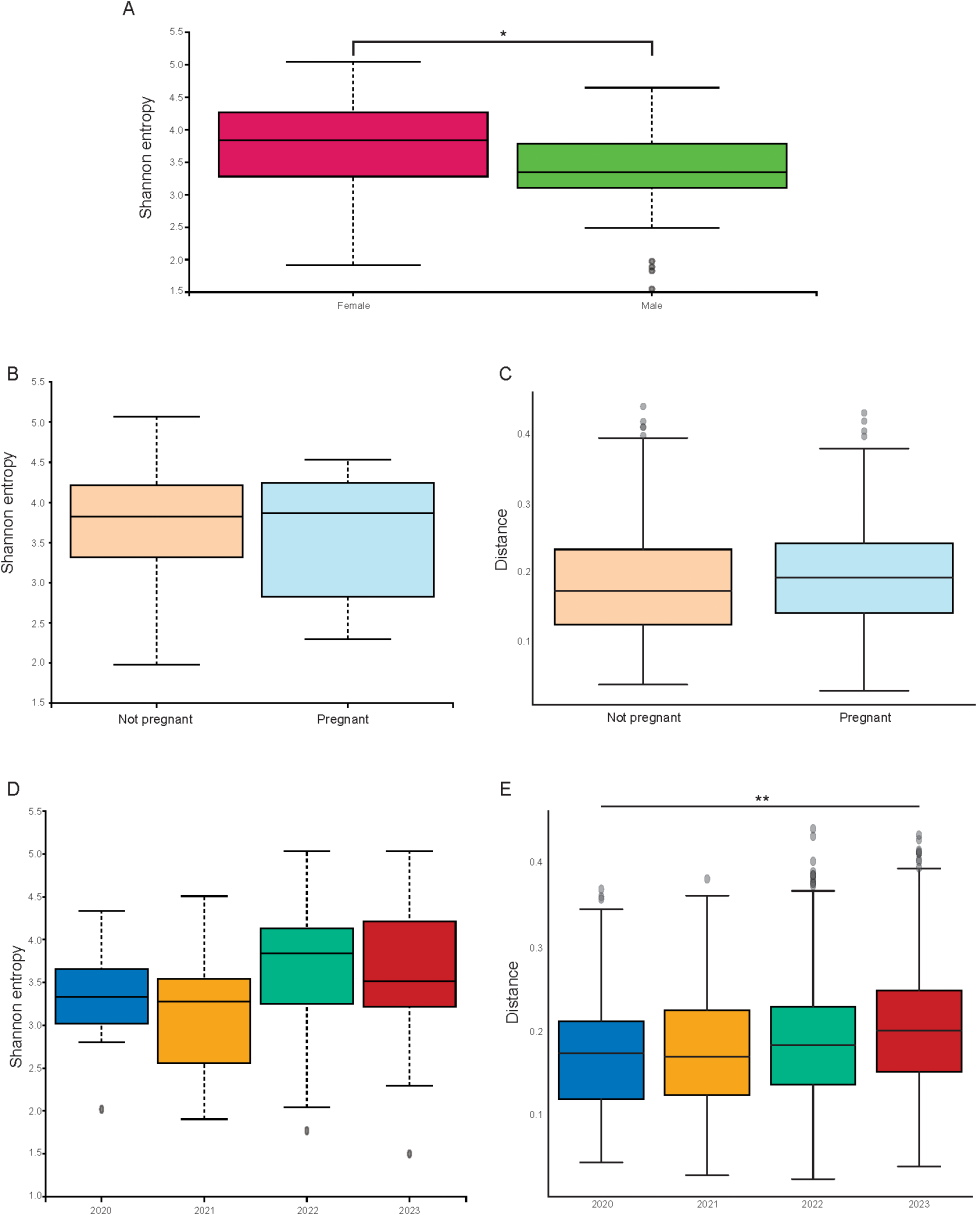
*P. sonoriensis* from Taos display within-group differences based on sex and year of capture. **(A)** Male mice from Taos have significantly reduced alpha diversity compared to females, Shannon p = 0.02. **(B)** There are no differences in the alpha diversity of pregnant and non-pregnant female mice from Taos. **(C)** There are no differences in the beta diversity of pregnant and non-pregnant female mice from Taos. **(D)** There are no differences in the alpha diversities of mice from Taos trapped in different years. **(E)** There are significant changes in the beta diversities of mice trapped in different years, weighted UNIFRAC p = 0.005. #*P ≤ 0.05, **P ≤ 0.01, ***P ≤ 0.001, ****P ≤ 0.0001.

Taos has the largest number of *P. sonoriensis* of any of the trapping locations and is the location that has been trapped the most times. We have trapped at the same time of year in the Taos location for four years beginning in 2020. When we analyzed the mice from Taos (**Figure 11**), we found that there are significant changes with regards to sex and year of capture (**Figures 11A, E**, sex: Shannon p = 0.02; year of capture: weighted UNIFRAC p = 0.005). There are no ethanol stored samples from Taos. This shift in the microbiome over time has been previously reported, even within the same individual organism (Hisada et al., 2015; Vandeputte et al., 2021; Fenn et al., 2023).

Taos also contained a large proportion of pregnant animals, which allowed us to specifically examine if there were any effects on the microbiome of these animals in response to pregnancy. As we had previously seen only minimal changes in the microbiome in relation to sex, we hypothesized that the significant physiological changes in pregnancy would result in larger shifts. Changes to the microbiome during pregnancy have also previously been reported in both lab-raised *Mus musculus* as well as humans (Koren et al., 2012; Elderman et al., 2018). However, when we compared pregnant and non-pregnant female mice, there were no significant changes to the microbiome (**Figures 11B, C**). Pregnancy alters the general immune response and would be expected to change the microbiome (Koren et al., 2012; Kareva, 2020). Potential effects of pregnancy on the immunology of *Peromyscus* and how this may differ from those of lab animals and other vertebrate animals should be further investigated.

### 3.7 Differential species abundance analysis reveals many possible associations

**Figure 12:**
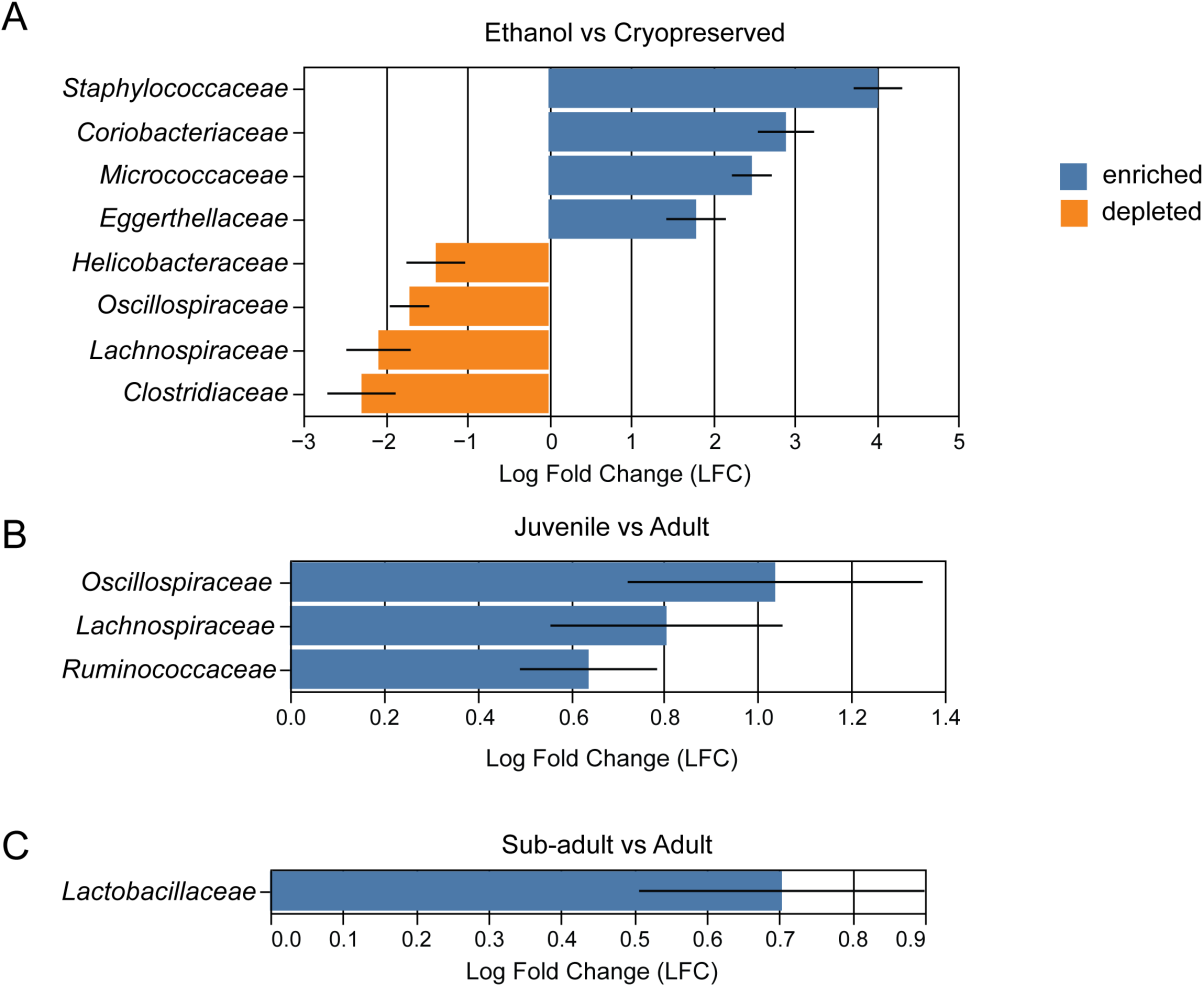
Differential abundance of bacteria based on storage conditions and age of mice. Analysis was performed using ANCOMBC. All analyses were performed at the family level. All changes were significant at the p = 0.05 threshold. **(A)** Differential bacterial abundance in ethanol stored samples compared to those that were cryopreserved. **(B)** Differential bacterial abundance in juvenile mice compared to adults. **(C)** Differential bacterial abundance in sub-adult mice compared to adults.

**Figure 13:**
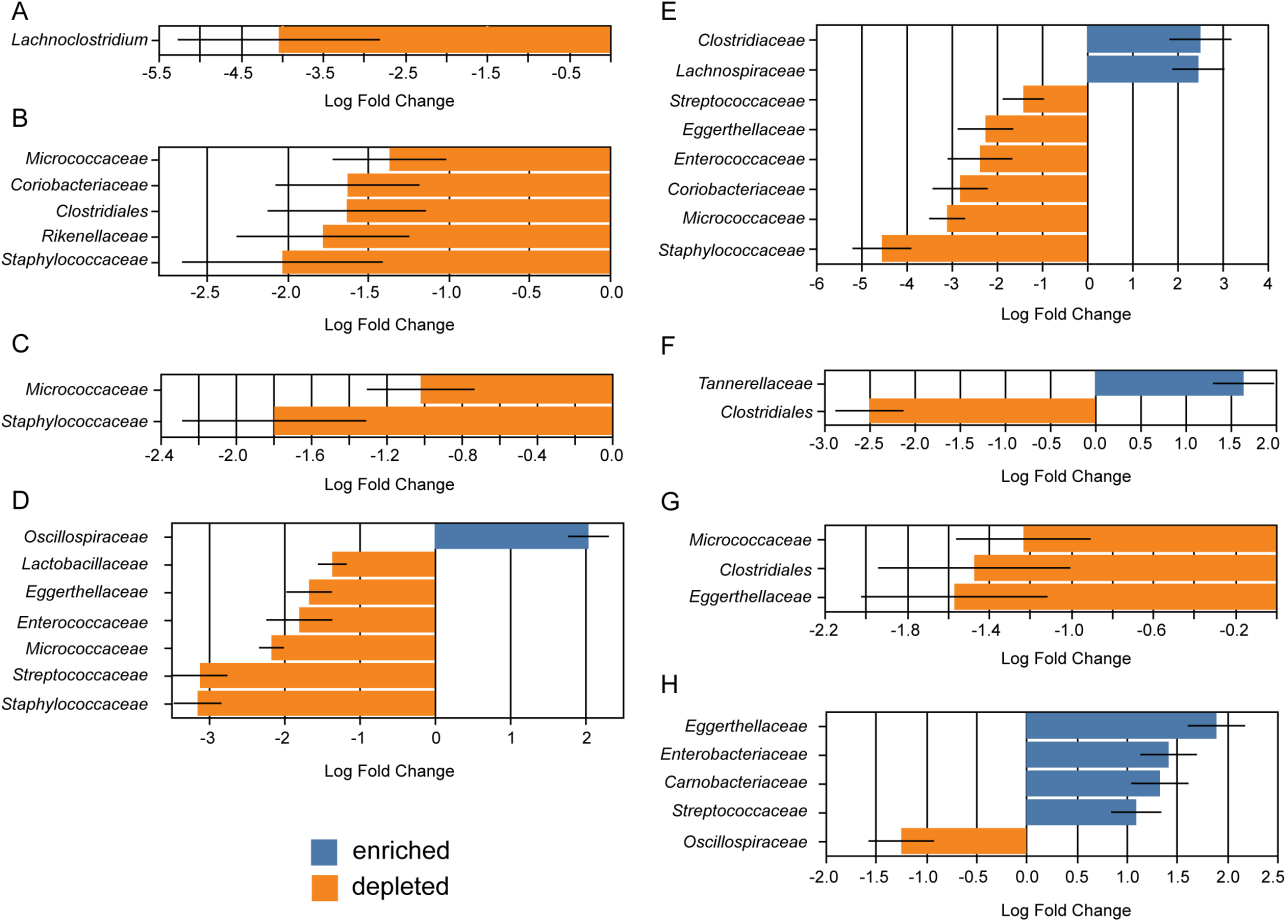
Differential abundance of bacteria based on trapping location. All graphs are the differential bacteria abundance in the Taos location compared to the indicated location. Analysis was performed using ANCOMBC. All analyses were performed at the family level unless otherwise indicated. **(A)** Differential bacterial abundance compared to the Blue Water location, shown at the genus level. **(B)** Differential bacterial abundance compared to the Cowels location, *Clostridiales* is a bacterial order. **(C)** Differential bacterial abundance compared to the El Malpais location. **(D)** Differential bacterial abundance compared to the ENMU location. **(E)** Differential bacterial abundance compared to the Hopewell location. **(F)** Differential bacterial abundance compared to the McGaffey location, *Clostridiales* is a bacterial order. **(G)** Differential bacterial abundance compared to the Pecos location, *Clostridiales* is a bacterial order. **(H)** Differential bacterial abundance compared to the Red Canyon location.

Our final analysis was an analysis of composition of microbiomes with bias correction (ANCOMBC) (Lin and Peddada, 2020; Lin et al., 2022; Lin and Peddada, 2024). The nature of ANCOMBC analysis requires the use of a “control” group for comparison. While none of these groups are actual controls, the group used for each comparison had been noted in analysis with more than two groups. We have indicated shifts in abundances at the family level followed with what is known about these bacteria in relation to the microbiome. Differential abundance plots of ethanol stored samples and mice divided by age can be seen in **Figure 12**. Differential abundance plots of mice from Taos compared to each named location are in **Figure 13**. All other differential abundance plots are in the supplemental information (**Supplementary Figures S1**, **S2**). Many of these bacterial families have not been well studied in rodents and most information about their possible physiological effects is from human association studies. There are essentially no studies in wild rodents proposing physiological relationships between these bacteria and the host. Due to this, many of the found associations are predicted to occur in humans, with some having been tested in laboratory animals.

There were various changes seen in the different ages of mice compared to adults. Juveniles had increased Oscillospiraceae, Lachnospiraceae, and Ruminoococcaceae. Oscillospiraceae have been found at increased levels in lean individuals compared to obese (Salazar-Jaramillo et al., 2024). Lachnospiraceae are producers of short chain fatty acids that have been tied to a wide variety of human diseases (Liang et al., 2020; Zeng et al., 2016; Vacca et al., 2020; Yilmaz et al., 2022; Zhang et al., 2022; Yu et al., 2025). Ruminococcaceae is related to sleep apnea in hypertensive humans and is important in the gut of lactose intolerant individuals. Ruminoococcaceae are also common in the rumen of ungulates (Creevey et al., 2014; Azcarate-Peril et al., 2021; Zhang et al., 2022). Sub-adult mice had increased Lactobacillaceae compared to adults, these bacteria are known to be important health mediators in the guts of mammals (Walter and O’Toole, 2023).

There were changes in bacterial abundances in Taos in relation to pregnancy and year of capture compared to 2020. Pregnant animals from Taos had decreases in *Ruminococcus* and *Enterococcus*, common commensals and pathogens (Dubin and Pamer, 2017; Dave et al., 2019). Taos animals caught in 2021 had an increase in Helicobacteraceae, shown to be increased in a high fat diet and *H. pylori* type species will dominate the stomach when present (Péré-Védrenne et al., 2017). Specimens caught in 2022 had increases of lactobacillus and clostridial species which are common in the human gut (Candeliere et al., 2023). Taos 2023 animals had decreased *Ruminoclostridium*, shown to have potential involvement in gut permeability and environmental sensitivity (Azcarate-Peril et al., 2021); *Kocuria*, a colonizer of plants and likely diet related (Stackebrandt et al., 1995), and *Adlercreutzia*, shown to be anti-inflammatory (Oñate et al., 2023).

For our locational analysis we selected the Taos group as it has the largest number of animals and is a primary ongoing study site. All locations had shifts in abundances when compared to Taos (**Table 4**).

**Table 4:** Differentially abundant bacteria at named locations compared to Taos.

Trapping location	Bacteria	Increased or decreased	Possible function
Red Canyon	Oscillospiraceae	increased	Increased in obesity (Salazar-Jaramillo et al., 2024)
	Eggerthellaceae	decreased	Increased in treatment resistant depression and individuals with hyperplastic intestinal polyps (Dong et al., 2022; Ruiz-Saavedra et al., 2024)
	Enterobacteriaceae	decreased	Known to be common, long term residents in human guts (Martinson et al., 2019)
	Carnobacteriaceae	decreased	Increased in individuals responsive to checkpoint therapy, decreased in cognitive decline (Yin et al., 2021; Komanduri et al., 2022)
	Streptococcaceae	decreased	Increased in high fat diet and COPD (Zeng et al., 2016; Bowerman et al., 2020)
McGaffey	Clostridiales	increased	Increased in psychiatric disorders, associated with decreased obesity (Li et al., 2020; Salazar-Jaramillo et al., 2024)
	Tannerellaceae	decreased	Increased in sleep deprivation (Zhang et al., 2023)
Hopewell	Staphylococcaceae	increased	Increased with ageing in humans (Odamaki et al., 2016)
	Micrococcaceae	increased	Common in soil, found in breast malignancy (Meng et al., 2018)
	Coriobacteriaceae	increased	Increased in neonatal dysbiosis, enriched in high fat diets resulting in increased colon tumors (Tang et al., 2024; Xiao et al., 2024)
	Enterococcaceae	increased	Common commensals and pathogens (Dubin and Pamer, 2017; Dave et al., 2019)
	Eggertherllaceae	increased	Increased in treatment resistant depression and individuals with hyperplastic intestinal polyps (Dong et al., 2022; Ruiz-Saavedra et al., 2024)
	Streptococcaceae	increased	Increased in high fat diet and COPD (Zeng et al., 2016; Bowerman et al., 2020)
	Clostridiaceae	decreased	Common in the human gut (Candeliere et al., 2023)
	Lachnospiraceae	decreased	Important producers of short chain fatty acids (Liang et al., 2020; Zeng et al., 2016; Vacca et al., 2020; Yilmaz et al., 2022; Zhang et al., 2022; Yu et al., 2025)
ENMU	Staphylococcaceae	increased	Increased with ageing in humans (Odamaki et al., 2016)
	Streptococcaceae	increased	Increased in high fat diet and COPD (Zeng et al., 2016; Bowerman et al., 2020)
	Micrococcaceaea	increased	Common in soil, found in breast malignancy (Meng et al., 2018)
	Enterococcaceeae	increased	Common commensals and pathogens (Dubin and Pamer, 2017; Dave et al., 2019)
	Eggerthellaceae	increased	Increased in treatment resistant depression and individuals with hyperplastic intestinal polyps (Dong et al., 2022; Ruiz-Saavedra et al., 2024)
	Lactobacillaceaeae	increased	Mediators of health in the mammalian gut (Walter and O’Toole, 2023)
	Oscillospiraceaeae	decreased	Increased in obesity (Salazar-Jaramillo et al., 2024)
El Malpais	Staphylococcacea	Increased	Increased with ageing in humans (Odamaki et al., 2016)
	Micrococcaceae	increased	Common in soil, found in breast malignancy (Meng et al., 2018)
Blue Water	Staphylococcacea	increased	Increased with ageing in humans (Odamaki et al., 2016)
	Micrococcaceae	increased	Common in soil, found in breast malignancy (Meng et al., 2018)
Pecos	Micrococcaceae	increased	Common in soil, found in breast malignancy (Meng et al., 2018)
	Clostridiales	increased	Increased in psychiatric disorders, associated with decreased obesity (Li et al., 2020; Salazar-Jaramillo et al., 2024)
	Eggerthellaceae	increased	Increased in treatment resistant depression and individuals with hyperplastic intestinal polyps (Dong et al., 2022; Ruiz-Saavedra et al., 2024)
Cowles	Micrococcaceae	increased	Common in soil, found in breast malignancy (Meng et al., 2018)
	Coriobacteriaceae	increased	Increased in neonatal dysbiosis, enriched in high fat diets resulting in increased colon tumors (Tang et al., 2024; Xiao et al., 2024)
	Clostridiales	increased	Increased in psychiatric disorders, associated with decreased obesity (Li et al., 2020; Salazar-Jaramillo et al., 2024)
	Staphylococcaceae	increased	Increased with ageing in humans (Odamaki et al., 2016)
	Rikenellaceae	increased	Associated with decreased adiposity and increased innate immune activation (Tavella et al., 2021; Yang et al., 2022)

Preservation in ethanol impacted composition of bacterial families compared to cryopreservation, with increases in Staphylococcaceae, Coriobacteriaceae, Microccacea, and Eggerthellacea. Ethanol preservation in our study resulted in decreases in Helicobacteraceae, Oscillospiraceae, Lachnoispiraceae, and Clostridiaceae. Overall ethanol preservation appears to favor detection of bacteria associated with inflammation and disease.

### 3.8 Multivariate analysis of the samples using GLMMS

Our previously described results for alpha diversity, beta diversity, and differential abundance have all been determined via pairwise testing with multiple testing correction. However, there is a growing appreciation that microbiome analysis may be improved by the inclusion of multivariant statistical testing (Sweeny et al., 2023). As we tested four theoretically independent variables alone and in combination, and as the microbiome samples of wild *P. sonoriensis* are predicted to highly heterogenous, we determined it would be beneficial to test multivariate statistical models for our diversity measures as well as differential abundance. These analyses have been performed using generalized mixed models as well as the extended process for multivariant analysis of ANCOMBC.

We first examined alpha diversity for differential impacts of our tested variables. When we tested the combinatorial effects, we found that by far the strongest effect exerted on alpha diversity was from the sample storage conditions. The impact was so great that there was no increased predictive value when any of the other variables were included in these multivariate models. Additionally, there were no issue with collinearity between the predictors, indicating that the different variables were independent from one another. Thus, our analysis showed that preservation method is the primary driver of observed variation in alpha diversity in this dataset. Full analysis results can be found in **Supplementary Table 2**.

Our analysis of combinatorial effects on beta diversity was the opposite. In this case, the more variables that were included in our beta diversity measures, the better the performance of our models became. For two of the PCoA axes used in this analysis, the full analysis had the lowest AIC-equivalent score, indicating the best performance. However, for axis 2, the ecological model, which includes only location, performed the best. Overall, these results indicate that different predictors contribute to distinct components of ecological variation, and integrating multiple covariates provides a more comprehensive representation of beta diversity patterns without implying statistical dependence among predictors. The specific performance of each model can be found in **Supplementary Figure 3**.

Lastly, we also performed a second round of differential abundance analysis using a version of the ANCOMBC analysis which incorporates GLMMs. In this instance we saw fewer differences between the samples over all when compared to our initial pairwise analysis. There were 6 differential taxa associated with ethanol preservation in the sample-level model, 6 in the host-level model, with the addition of sex and age. There were 7 differential taxa in the ecological-level model, which added location. And there were 7 in the full model, which included all covariates. This indicates that host variables had minimal influence on differential abundance, whereas incorporating ecological context slightly increased the number of detected taxa. These results are summarized in **Supplementary Table 3**. Overall, the associations with ethanol exposure were robust and remained significant across models, suggesting that ethanol preservation is the strongest predictor of differential taxa and is fully independent of all other variables.

## 4 Discussion

This multisite study of wild *Peromyscus sonoriensis* in the Southwest United States provides new insights into the effect of location, habitat, sex, and age on the composition of the gut microbiome. While other studies have compared wild and lab-reared *Peromyscus*, none was performed at this geographic scale, or examined mice from the same biomes included in our study (Coolon et al., 2010; Baxter et al., 2015; Schmidt et al., 2019; Milovic et al., 2020; Scheepers et al., 2020; Diaz et al., 2023; Mistrick et al., 2024; Wald, 2024; Brinkerhoff et al., 2025). With the increased reference data for the *Peromyscus sonoriensis* microbiome provided by this study, these data can be used for comparison to other genera as well. We have also demonstrated the usefulness of archival, museum-stored samples for increasing the number and scope of samples available for microbiome studies.

The most interesting findings of our study may relate not to differences between these mice, but instead to how similar they are to one another. We found that overall, mice from the same or similar trapping locations have highly similar microbiomes, with minimal differences in either alpha or beta diversity. This was surprising as there are strong effects on the intestinal microbiome of humans mediated by the biological conditions of the host (Koren et al., 2012; Yatsunenko et al., 2012; Markle et al., 2013; Leite et al., 2021). However, in our overall sample population, various biological attributes (sex, age, and pregnancy) had minimal effects on the microbiome, with only location of capture and sample storage conditions having significant effects overall.

Multiple biological characteristics and conditions have been documented to drive alterations in the gut microbiome, such as sex, age, and pregnancy, yet we found no overall differences in relation to those characteristics. This is possibly due to high individual variation overwhelming the effects of these biological attributes. Alternatively, physiological differences in these groups (sex, age, pregnancy status) may be less distinct in *Peromyscus* mice compared to other mammals. The effects of androgens on the immune system are thought to be the main drivers of differences in the microbiome between different aged organisms, as well as the differences between organisms of different sexes (Markle et al., 2013; Trigunaite et al., 2015; Kim, 2022). To our knowledge, there is no data on the effects of androgens on the immune milieu of *Peromyscus* mice. There is evidence that the estrus cycle has an effect on the microbiome of the gut in various mammals, however the effects in mice are still controversial (Wallace et al., 2018; Comizzoli et al., 2021; Zhang et al., 2021; Sisk-Hackworth et al., 2023; Matějková et al., 2024). Research indicates that female *Peromyscus* mice are often more social and active during their heat (Dewsbury et al., 1977; Cushing, 1985; Karelina et al., 2010). This makes it possible that the majority of our female mice are in similar stages of their cycle, and that this could explain some of the variation or lack thereof in our samples.

This lack of significant differences across the study populations led us to isolate different groups of mice for further testing, but the general trend was that within a sample from a given location there would be few, if any, significant differences detected. While individual mice host diverse microbial communities, at the population level *P. sonoriensis* appear to share relatively similar microbiomes. Further studies of the gut microbiomes of other *Peromyscus* species, and other *P. sonoriensis* populations in different areas of North America, would be required to determine if this a feature of *Peromyscus* mice broadly, or a specific characteristic of those found in the various ecoregions of New Mexico.

Location is a significant contributor to the composition of the microbiome in these animals as demonstrated in the bar taxa plots (**Figure 3**), PCA plots (**Figure 4**), and analyses of alpha and beta diversities (**Figure 4**; **Tables 3**, **4**). Pairwise comparisons indicate that ecoregion variation is likely involved, as locations that are distant from each other but share similar ecoregions are more similar than those that are from distinct but geographically close ecoregions (i.e., Blue Water and McGaffey versus Blue Water and Pecos). There is also the possibility that differences in altitude play a prominent role, as the hypoxic nature of the mammalian gut is strongly interactive with the bacterial species present, and environmental factors are known to be among the strongest drivers of the microbiome composition of all animals (Maritan et al., 2024; Zheng et al., 2015). These locational and ecological based effects are possible driven by both differences in the mouse diet as well as differences in bacteria present in the environment.

*Peromyscus* are generalists that eat a large amount of plant material as well as small animals such as arthropods (Jameson, 1952; Wolff et al., 1985; Vázquez et al., 2004; Bedford and Hoekstra, 2015). Different ecoregions will have different plant and prey species available as food sources, resulting both in different microbes available from the diet to colonize the gut, as well as different species of bacteria being adaptively selected as part of the rodent digestive processes. Dietary changes have been shown to rapidly result in consistent changes to the gut microbiome which can even be tracked with stable isotopes. These differences occur in many different animals, including mice, and so are highly likely to occur in *Peromyscus* species as well (Muegge et al., 2011; Wang et al., 2014; Maurice et al., 2015; Kwon and Seong, 2021). The microbiome is also recognized to perform important dietary functions in mammals, and these functions can be predicted by the presence or absence of different microbial communities (Muegge et al., 2011). Combined with additional research into the functions of the various microbes found in the *Peromyscus* gut, the data set published here could then be utilized to predict dietary differences between *P. sonoriensis* in different environments, or between various related cricetid rodent species. *Peromyscus sonoriensis* move throughout their environments in both terrestrial and arboreal spaces (Wolff and Hurlbutt, 1982; Graves et al., 1988), and different biomes have different compliments of plant species which have highly specific microbial communities (Berg et al., 2016) as well as different biomes having distinct soil microbiomes (Vasar et al., 2022). These differential pro- and eukaryotic microbes are potential colonizers of the mammalian gut. Location as a driver of diversity in the microbiome is also documented in humans, and has been suggested to be driven by diet (Yatsunenko et al., 2012; Gupta et al., 2017).

The effects of the environment on the microbiome can also vary over time. We found in that mice trapped in the same location at different times of year have significantly altered microbiomes (McGaffey **Figures 10C, D**). These differences were noted between late summer and late autumn/early winter. As such, there were likely differences in food availability as well mouse activity. Seasonality has also previously been reported to affect the mouse microbiome (Maurice et al., 2015). In addition to finding effects related to seasonality, out data also show year-over-year alterations in the composition of the gut microbiome of *P. sonoriensis.* We have collected animals from the Taos location every year for four years, each year in August. When we analyzed the microbiomes of the mice collected in each year, we found that there were no changes in alpha diversity but significant alterations in the beta diversity. This indicates that while the numbers and proportions of bacteria present were remaining similar, there were alterations in the specific bacterial species that were present. This indicates that there are likely different selective pressures in the different trapping years, resulting in changes in the bacterial community. This could be due to changes in climate, food availability, or simple community drift over time. Temporal shifts in the microbiome are also a well-known phenomenon, and have been described even within the same individual organism (Hisada et al., 2015; Vandeputte et al., 2021; Fenn et al., 2023).

We were fortunate to have access to a large amount of museum archival rodent samples in this study. However, a fraction of these samples were preserved in 95% ethanol instead of cryopreserved. Because of this, we compared the microbiomes between the different storage conditions in our study and found an even stronger relationship with storage conditions than location of capture.

It is common in the preservation of whole mammal carcasses to store them in a high percentage of ethanol instead of using cryopreservation. This has the advantage of being more cost effective, as well as allowing storage of the sample in standard, room temperature conditions compared to the more difficult task of supplying a large number of samples with space in cold storage. However, it has been seen that storage in ethanol alters the microbes that are detected using molecular methods (Marotz et al., 2021; Li et al., 2023). This has held true in our samples as well. While nucleic acid is highly stabled in ethanol at high concentrations, it has been proposed in previous investigations of gastric helminths that degradation of source DNA may play a role in variable detection (Greiman et al., 2018; Marquina et al., 2021). When compared to samples that were cryopreserved, those that were stored in ethanol had significant alterations in their beta diversities and had significant reductions in their alpha diversities. These data indicate that ethanol storage results in a reduction in the variety of bacteria detected. When differential abundance analysis was performed on the bacterial families present in the samples, the ethanol preserved samples had preferential detection of bacteria that have been shown to be associated with various inflammatory phenotypes in lab animals and humans (**Figure 12A**).

As mentioned above, we were surprised at the lack of differences that were found in the microbiomes of mice of different biological categories, and the similarities between male and female mice, and between pregnant and non-pregnant females were particularly striking. As mentioned previously, it has been established that sex is a common driver of differences in the microbiomes of animals and humans (Markle et al., 2013; Kim, 2022). However, we found that there was no significant difference between male and female mice in this data set, and only Taos and ENMU mice had minor differences in alpha diversity between male and female mice after division by location.

Sex differences in the microbiome are thought to be derived from the differential effects of androgens and estrogens on the immune system, with androgens being generally immune suppressive (Trigunaite et al., 2015). This immune suppression is thought to allow differential outgrowth of bacteria in the guts of male mammals compared to the more immune reactive milieu that is present in the female gut. This lack of difference between male and female mice in this population indicates that there is a possible difference in the functions of these sexual hormones in *Peromyscus* mice compared to other mammals. This would require further characterization of the immune responses and inflammatory cascade as it occurs in *Peromyscus* mice. This research would also be helpful for the use of these animals as laboratory models, as many of their uses involve physiologies related to inflammation.

Pregnancy has also been previously established as a microbiome altering event in humans and laboratory animal models (Koren et al., 2012; Elderman et al., 2018). Pregnancy is a dramatic hormonal process that alters almost all the mother’s physiology, and it is particularly influential on the immune response (Kareva, 2020). Thus, we hypothesized that the general downregulation in immune responsiveness that occurs would result in a temporarily altered microbiome. However, there was no discernable alteration in the microbiome of the pregnant animals compared to the non-pregnant females, when we examined pregnant and non-pregnant female animals from Taos.

This lack of difference in the microbiome of the female mice during pregnancy brings up similar questions to those already raised by the lack of difference between the male and female mice. It is already well understood that pregnancy in small rodents is quite distinct from that experienced by humans, with a neonate mouse being the equivalent to a third trimester fetus in a human (Hill, 2025). But despite these differences, pregnancy is noted to affect the microbiomes of both humans and lab reared mice (Koren et al., 2012; Elderman et al., 2018). Our data would indicate that some of these physiological changes that we know occur during pregnancy in humans and *Mus musculus* either do not occur or do not occur to the same degree in wild *P. sonoriensis;* thus, additional research is needed, specifically into the immunological effects of pregnancy in *Peromyscus* species.

The sample set used in this research is highly complex, and due to the nature of wild animal collections is also very heterogenous. Due to this high level of variance, we determined that it would be beneficial to test multivariant statistical approaches in addition to our pair-wise comparisons with multiple testing correction. We tested generalized mixed models for alpha diversity, beta diversity, and differential taxa abundance at the family level. Interestingly we found that there were different impacts of this testing on each of our three measures.

Alpha diversity was dominated by a single variable, ethanol preservation. Beta diversity models were the strongest when all variables were included. And most differential abundance could be explained by ethanol storage, but the inclusion of some additional variables resulted in additional differential taxa. These results demonstrate the unpredictability of these analyses, and that inclusion of additional variables does not always increase the predictive power of a model. These analyses also showed commonalities and differences with out pair-wise comparisons. It is not surprising based on our initial analysis that ethanol preservation dominated our alpha diversity measures, as in our initial tests this variable had the strongest effect by far. This also supports previous data showing that storage conditions play an important role in microbiome composition. It was surprising, however, that storage condition played such a dominant role in ANCOMBC. We found in our pair-wise comparison that there were strong effects of many different variables; some that had no ethanol stored samples among them. This indicates that such a strong variable may mask the effects of other weaker variables in these analyses. It was also no surprise that each of our variables was important to beta diversity. Location and storage conditions had both been found already to have significant effects on the beta diversity metrics of these mice. Those variables that didn’t already have a significant association were also known in other organisms and publications to be important drivers of beta diversity as well. So, the fact that each of these variables would contain important information about beta diversity is consistent.

With increasing interest in the microbiomes of wild animals and less controlled populations of organisms, the authors would encourage the further use and development of multivariant models specifically for microbiome analysis. The inclusion of these models and statistics serve to better describe the full and combinatorial effects of the increased diversity that is seen in these sample sets.

In addition to the various comparisons of the different alpha and beta diversity metrics, we also analyzed the differential abundance of bacteria in our samples at the family level using analysis of composition with bias correction (ANCOMBC). We found that even when a variable did not have a significant effect on diversity, there were still some bacterial families that were differentially abundant (**Supplementary Figures 2**, **3**).

Variables that did have significant alterations in the alpha and beta diversities had a large number of differentially abundant bacterial families at the 0.05 significant level. Most studies into the possible physiological impacts of commensal microbes have been done as observational studies in humans, with a few having been experimentally examined in gnotobiotic or germ-free mouse models. This makes it difficult to accurately hypothesize what changes may be happening at the organismal level when a given family of bacteria is increased or decreased.

There are few studies examining the differential abundance of bacterial taxa in wild-caught rodents, and as mentioned in the results section none that examine the impacts of these taxa on the physiology of the rodents, but some have examined the effect of urbanization and human land utilization on the microbiomes of *P. maniculatus (*Diaz et al., 2023), the closest relative of *P. sonoriensis*, and several rat species: *Rattus rattus, Rattus norvegicus, Sundamys muelleri*, and *Suncus murinus (*Giacomini et al., 2025). Increasing urbanization resulted in decreases in species of *Micrococcal, Staphylococcal, Lachnospiral*, and *Oscillospiral* bacteria in *P. maniculatus (*Diaz et al., 2023). We saw increases in Microccus and Staphylococcus and reductions in Lachnospira and Oscillospira in ethanol storage compared to cryopreservation. There was an increase in *Lachnospira* and *Oscillospira* in juveniles compared to adults. Micrococcal and staphylococcal species were reduced at many trapping locations compared to the Taos site. *Lachnospira* were increased in the Taos location compared to McGaffey. *Oscillospira* were decreased at the Taos location compared to Red Canyon. The Taos site is close to multiple human habitations and would have the most intensive human activity of any of our locations.

Lactobacilli (in *R. norvegicus*) and clostridial species (in *R. norvegicus* and *S. murinus*) were previously shown to be increased in less developed areas (Giacomini et al., 2025). In our animals Lactobacilaceae were increased in sub-adult compared to adult mice, and decreased in Taos compared to ENMU. Clostridial bacteria were decreased after ethanol storage and increased at Hopewell compared to Taos, while the Order Clostridiales was decreased in many locations compared to Taos. Increased land use intensity has been shown to decrease species of *Lachnospira, Oscillobacter, Rickenella* (*all in R. norvegicus*), and *Enterobacter* (*S. murinus*) *(*Giacomini et al., 2025). In our data, *Rickenella* decreased at Cowles compared to Taos and enterobacter was increased at Red Canyon compared to Taos. Increased land use intensity has been shown to increase the prevalence of lactobacillus, clostridia, ruminococcus, staphylococcus, and helicobacter (all in *R. norvegicus*) (Giacomini et al., 2025). Our analysis showed increased ruminococcus in juvenile mice compared to adults, and helicobacter was reduced after storage in ethanol.

In conclusion, while the gut microbiomes of *P. sonoriensis* mice from New Mexico are highly diverse and location appears to drive microbiome differences, several differences found in studies of other animal populations and humans are not present in this study. The lack of gut microbiome differences due to sex or pregnancy are especially notable. These data provide a reference for future research of *Peromyscus* species including comparisons to wild mice populations from other regions and environments.

## Data Availability

The datasets presented in this study can be found in online repositories. The names of the repository/repositories and accession number(s) can be found below: https://www.ncbi.nlm.nih.gov/, PRJNA1292242/.

## References

[B1] Anderson M. J. (2001). A new method for non-parametric multivariate analysis of variance. Austral Ecol. 26, 32–46. doi: 10.1046/j.1442-9993.2001.01070.x. PMID: 41717205

[B2] Arquilla A. M. Wilson K. M. Razak K. A. Saltzman W. (2023). Fatherhood increases attraction to sensory stimuli from unrelated pups in male California mice, Peromyscus californicus. Anim. Behav. 198, 131–140. doi: 10.1016/j.anbehav.2023.02.001. PMID: 41940325

[B3] Azcarate-Peril M. A. Roach J. Marsh A. Chey W. D. Sandborn W. J. Ritter A. J. . (2021). A double-blind, 377-subject randomized study identifies Ruminococcus, Coprococcus, Christensenella, and Collinsella as long-term potential key players in the modulation of the gut microbiome of lactose intolerant individuals by galacto-oligosaccharides. Gut Microbes 13, 1957536. doi: 10.1080/19490976.2021.1957536. PMID: 34365905 PMC8354614

[B4] Banther-McConnell J. K. Suriyamongkol T. Goodfellow S. M. Nofchissey R. A. Bradfute S. B. Mali I. (2024). Distribution and prevalence of Sin Nombre hantavirus in rodent species in eastern New Mexico. PLoS One 19, e0296718. doi: 10.1371/journal.pone.0296718. PMID: 38236803 PMC10796054

[B5] Bautista N. M. Herrera N. D. Shadowitz E. Wearing O. H. Cheviron Z. A. Scott G. R. . (2024). Local adaptation, plasticity, and evolved resistance to hypoxic cold stress in high-altitude deer mice. Proc. Natl. Acad. Sci. 121, e2412526121. doi: 10.1073/pnas.2412526121. PMID: 39352929 PMC11474095

[B6] Baxter N. T. Wan J. J. Schubert A. M. Jenior M. L. Myers P. Schloss P. D. (2015). Intra- and interindividual variations mask interspecies variation in the microbiota of sympatric Peromyscus populations. Appl. Environ. Microbiol. 81, 396–404. doi: 10.1128/aem.02303-14. PMID: 25362056 PMC4272734

[B7] Bedford N. L. Hoekstra H. E. (2015). Peromyscus mice as a model for studying natural variation. eLife 4, e06813. doi: 10.7554/elife.06813. PMID: 26083802 PMC4470249

[B8] Berg G. Rybakova D. Grube M. Köberl M. (2016). The plant microbiome explored: implications for experimental botany. J. Exp. Bot. 67, 995–1002. doi: 10.1093/jxb/erv466. PMID: 26547794 PMC5395086

[B9] Bolyen E. Rideout J. R. Dillon M. R. Bokulich N. A. Abnet C. C. Al-Ghalith G. A. . (2019). Reproducible, interactive, scalable and extensible microbiome data science using QIIME 2. Nat. Biotechnol. 37, 852–857. doi: 10.1038/s41587-019-0209-9. PMID: 31341288 PMC7015180

[B10] Botten J. Mirowsky K. Kusewitt D. Bharadwaj M. Yee J. Ricci R. . (2000). Experimental infection model for Sin Nombre hantavirus in the deer mouse (Peromyscus maniculatus). PNAS 97, 10578–10583. doi: 10.1073/pnas.180197197. PMID: 10973478 PMC27067

[B11] Bowerman K. L. Knowles S. C. L. Bradley J. E. Baltrūnaitė L. Lynch M. D. J. Jones K. M. . (2021). Effects of laboratory domestication on the rodent gut microbiome. ISME Commun. 1, 1–14. doi: 10.1038/s43705-021-00053-9. PMID: 36747007 PMC9723573

[B12] Bowerman K. L. Rehman S. F. Vaughan A. Lachner N. Budden K. F. Kim R. Y. . (2020). Disease-associated gut microbiome and metabolome changes in patients with chronic obstructive pulmonary disease. Nat. Commun. 11, 5886. doi: 10.1038/s41467-020-19701-0. PMID: 33208745 PMC7676259

[B13] Bradley R. Francis J. Platt R. I. Soniat T. Alvarez D. Lindsey L. (2019). Mitochondrial DNA sequence data indicate evidence for multiple species within Peromyscus maniculatus ( Special Publications, The Museum Texas Tech. University), 1–59.

[B14] Brinkerhoff R. J. Pandian J. Leber M. Hauser I. D. Gaff H. D. (2025). Impacts of tick parasitism on the rodent gut microbiome. Microorganisms 13, 888. doi: 10.3390/microorganisms13040888. PMID: 40284724 PMC12029286

[B15] Candeliere F. Musmeci E. Amaretti A. Sola L. Raimondi S. Rossi M. (2023). Profiling of the intestinal community of Clostridia: taxonomy and evolutionary analysis. Microbiome Res. Rep. 2, 13. doi: 10.20517/mrr.2022.19. PMID: 38047279 PMC10688793

[B16] Chen L. Wang D. Garmaeva S. Kurilshikov A. Vila A. V. Gacesa R. . (2021). The long-term genetic stability and individual specificity of the human gut microbiome. Cell. 184, 2302–2315.e12. doi: 10.1016/j.cell.2021.03.024. PMID: 33838112

[B17] Chen Y. H. Yeung F. Lacey K. A. Zaldana K. Lin J. D. Bee G. C. W. . (2023). Rewilding of laboratory mice enhances granulopoiesis and immunity through intestinal fungal colonization. Sci. Immunol. 8, eadd6910. doi: 10.1126/sciimmunol.add6910. PMID: 37352372 PMC10350741

[B18] Chi L. H. Burrows A. D. Anderson R. L. (2022). Can preclinical drug development help to predict adverse events in clinical trials? Drug Discov. Today 27, 257–268. doi: 10.1016/j.drudis.2021.08.010. PMID: 34469805

[B19] Colins H. H. (1923). Studies of the pelage phases and of the nature of color variations in mice of the genus Peromyscus. J. Exp. Zool. 38, 45–107. doi: 10.1002/jez.1400380103, PMID: 41531421

[B20] Comizzoli P. Power M. L. Bornbusch S. L. Muletz-Wolz C. R. (2021). Interactions between reproductive biology and microbiomes in wild animal species. Anim. Microbiome 3, 87. doi: 10.1186/s42523-021-00156-7. PMID: 34949226 PMC8697499

[B21] Coolon J. D. Jones K. L. Narayanan S. Wisely S. M. (2010). Microbial ecological response of the intestinal flora of Peromyscus maniculatus and P. leucopus to heavy metal contamination. Mol. Ecol. 19, 67–80. doi: 10.1111/j.1365-294X.2009.04485.x, PMID: 20331771

[B22] Creevey C. J. Kelly W. J. Henderson G. Leahy S. C. (2014). Determining the culturability of the rumen bacterial microbiome. Microb. Biotechnol. 7, 467–479. doi: 10.1111/1751-7915.12141. PMID: 24986151 PMC4229327

[B23] Cushing B. S. (1985). A comparison of activity patterns of estrous and diestrous prairie deer mice, Peromyscus maniculatus bairdi. J. Mammology 66, 136–139. doi: 10.2307/1380967. PMID: 39964225

[B24] Dave V. P. Pathengay A. Braimah I. Z. Panchal B. Sharma S. Pappuru R. R. . (2019). Enterococcus endophthalmitis: clinical settings, antimicrobial susceptibility, and management outcomes. Retina. 10, 898–902. doi: 10.1097/IAE.0000000000002462, PMID: 30681640

[B25] Davis E. C. Castagna V. P. Sela D. A. Hillard M. A. Lindberg S. Mantis N. J. . (2022). Gut microbiome and breast-feeding: implications for early immune development. J. Allergy Clin. Immunol. 150, 523–534. doi: 10.1016/j.jaci.2022.07.014. PMID: 36075638 PMC9463492

[B26] Dewsbury D. A. Estep D. Q. Lanier D. L. (1977). Estrous cycles of nine species of muroid rodents. J. Mammology 58, 89–92. doi: 10.2307/1379735. PMID: 557523

[B27] Diaz J. Redford K. H. Reese A. T. (2023). Captive and urban environments are associated with distinct gut microbiota in deer mice (Peromyscus maniculatus). Biol. Lett. 19, 20220547. doi: 10.1098/rsbl.2022.0547. PMID: 36883780 PMC9994099

[B28] Dice L. R. (1936). Age variation in Peromyscus maniculatus gracilis. J. Mammal. 17, 55–57. doi: 10.2307/1374552, PMID: 39421808 PMC11484079

[B29] Dice L. R. Bradley R. M. (1942). Growth in the deer-mouse, Peromyscus maniculatis. J. Mammology 23, 416–427. doi: 10.2307/1375053, PMID: 39421620

[B30] Dong Z. Shen X. Hao Y. Li J. Xu H. Yin L. . (2022). Gut microbiome: a potential indicator for predicting treatment outcomes in major depressive disorder. Front. Neurosci. 16. doi: 10.3389/fnins.2022.813075, PMID: 35937875 PMC9354493

[B31] Dubin K. Pamer E. G. (2017). Enterococci and their interactions with the intestinal microbiome. Microbiol. Spectr. 5. doi: 10.1128/microbiolspec.bad-0014-2016. PMID: 29125098 PMC5691600

[B32] Elderman M. Hugenholtz F. Belzer C. Boekschoten M. de Haan B. de Vos P. . (2018). Changes in intestinal gene expression and microbiota composition during late pregnancy are mouse strain dependent. Sci. Rep. 8, 10001. doi: 10.1038/s41598-018-28292-2. PMID: 29968760 PMC6030191

[B33] Faith D. P. (1992). Conservation evaluation and phylogenetic diversity. Biol. Conserv. 61, 1–10. doi: 10.1016/0006-3207(92)91201-3

[B34] Fan S. Guo W. Xiao D. Guan M. Liao T. Peng S. . (2023). Microbiota-gut-brain axis drives overeating disorders. Cell Metab. 35, 2011–2027.e7. doi: 10.1016/j.cmet.2023.09.005. PMID: 37794596

[B35] Fenn J. Taylor C. Goertz S. Wanelik K. M. Paterson S. Begon M. . (2023). Discrete patterns of microbiome variability across timescales in a wild rodent population. BMC Microbiol. 23, 87. doi: 10.1186/s12866-023-02824-x. PMID: 36997846 PMC10061908

[B36] Giacomini A. Lakim M. B. Tuh F. Y. Y. Hitchings M. Consuegra S. Webster T. U. . (2025). Host-microbiome associations of native and invasive small mammals across a tropical urban–rural ecotone. Mol. Ecol. 34, e17782. doi: 10.22541/au.173720389.93870617/v1. PMID: 40289691 PMC12100590

[B37] Goodfellow S. M. Nofchissey R. A. Morley V. J. Coan K. E. Schwalm K. C. Cook J. A. . (2024). Genome sequencing identifies “Limestone Canyon virus” as Montaño virus (Hantaviridae: Orthohantavirus montanoense) circulating in brush deermice in New Mexico. NPJ Viruses 2, 11. doi: 10.1038/s44298-024-00016-6. PMID: 40295699 PMC11721154

[B38] Goodfellow S. M. Nofchissey R. A. Schwalm K. C. Cook J. A. Dunnum J. L. Guo Y. . (2021). Tracing transmission of Sin Nombre virus and discovery of infection in multiple rodent species. J. Virol. 95, e0153421. doi: 10.1128/jvi.01534-21. PMID: 34549977 PMC8577387

[B39] Goodfellow S. M. Nofchissey R. A. Ye C. Banther-McConnell J. K. Suriyamongkol T. Cook J. A. . (2025). A human pathogenic hantavirus circulates and is shed in taxonomically diverse rodent reservoirs. PLoS Pathog. 21, e1012849. doi: 10.1371/journal.ppat.1012849. PMID: 39836687 PMC11785344

[B40] Graves S. Maldonado J. Wolff J. O. (1988). Use of ground and arboreal microhabitats by Peromyscus leucopus and Peromyscus maniculatus. Can. J. Zool. 66, 277–278. doi: 10.1139/z88-040, PMID: 34819996

[B41] Greenbaum I. Honeycutt R. Chirhart S. (2019). Taxonomy and phylogenetics of the Peromyscus maniculatus species group ( Special Publications, The Museum Texas Tech. University), 559–575.

[B42] Greiman S. E. Cook J. A. Tkach V. V. Hoberg E. P. Menning D. M. Hope A. G. . (2018). Museum metabarcoding: a novel method revealing gut helminth communities of small mammals across space and time. Int. J. For. Parasitol. 48, 1061–1070. doi: 10.1016/j.ijpara.2018.08.001. PMID: 30315762

[B43] Greyson-Gaito C. J. Bartley T. J. Cottenie K. Jarvis W. M. C. Newman A. E. M. Stothart M. R. (2020). Into the wild: microbiome transplant studies need broader ecological reality. Proc. R. Soc. B. Biol. Sci. 287, 20192834. doi: 10.1098/rspb.2019.2834. PMID: 32097591 PMC7062022

[B44] Gupta V. K. Paul S. Dutta C. (2017). Geography, ethnicity or subsistence-specific variations in human microbiome composition and diversity. Front. Microbiol. 8. doi: 10.3389/fmicb.2017.01162, PMID: 28690602 PMC5481955

[B45] Halko N. Martinsson P. G. Shkolnisky Y. Tygert M. (2011). An algorithm for the principal component analysis of large data sets. SIAM J. Sci. Computing. 35. doi: 10.1137/100804139

[B46] Hamady M. Lozupone C. Knight R. (2010). Fast UniFrac: facilitating high-throughput phylogenetic analyses of microbial communities including analysis of pyrosequencing and PhyloChip data. ISME J. 4, 17–27. doi: 10.1038/ismej.2009.97. PMID: 19710709 PMC2797552

[B47] Hill M. (2025). Mouser timeline detailed. Available online at: https://embryology.med.unsw.edu.au/embryology/index.php?title=Mouse_Timeline_Detailed.

[B48] Hird S. M. (2017). Evolutionary biology needs wild microbiomes. Front. Microbiol. 8. doi: 10.3389/fmicb.2017.00725. PMID: 28487687 PMC5404107

[B49] Hisada T. Endoh K. Kuriki K. (2015). Inter- and intra-individual variations in seasonal and daily stabilities of the human gut microbiota in Japanese. Arch. Microbiol. 197, 919–934. doi: 10.1007/s00203-015-1125-0. PMID: 26068535 PMC4536265

[B50] Horvath S. Haghani A. Zoller J. A. Naderi A. Soltanmohammadi E. Farmaki E. . (2022). Methylation studies in Peromyscus: aging, altitude adaptation, and monogamy. GeroScience 44, 447–461. doi: 10.1007/s11357-021-00472-5. PMID: 34698996 PMC8810952

[B51] Ippolito M. M. Denny J. E. Langelier C. Sears C. L. Schmidt N. W. (2018). Malaria and the microbiome: a systematic review. Clin. Infect. Dis. 67, 1831–1839. doi: 10.1093/cid/ciy374. PMID: 29701835 PMC6260159

[B52] Jameson E. W. (1952). Food of deer mice, Peromyscus maniculatus and P. Boylei in the Northern Sierra Nevada, California. J. Mammal. 33, 50–60. doi: 10.2307/1375640, PMID: 29296531 PMC5739552

[B53] Kalkvik H. M. Stout I. J. Doonan T. J. Parkinson C. L. (2012). Investigating niche and lineage diversification in widely distributed taxa: phylogeography and ecological niche modeling of the Peromyscus maniculatus species group. Ecography 35, 54–64. doi: 10.1111/j.1600-0587.2011.06994.x. PMID: 41940437

[B54] Karelina K. Walton J. C. Weil Z. M. Norman G. J. Nelson R. J. DeVries A. C. (2010). Estrous phase alters social behavior in a polygynous but not a monogamous Peromyscus species. Horm. Behav. 58, 193–199. doi: 10.1016/j.yhbeh.2010.03.022. PMID: 20382149

[B55] Kareva I. (2020). Immune suppression in pregnancy and cancer: parallels and insights. Transl. Oncol. 13, 100759. doi: 10.1016/j.tranon.2020.100759. PMID: 32353791 PMC7191218

[B56] Kim N. (2022). “ Sex difference of gut microbiota,” in Sex/gender-specific medicine in the gastrointestinal diseases. Ed. Kim N. ( Springer Nature, Singapore), 363–377. Available online at: doi: 10.1007/978-981-19-0120-1_22. PMID:

[B57] Komanduri M. Savage K. Lea A. McPhee G. Nolidin K. Deleuil S. . (2022). The relationship between gut microbiome and cognition in older Australians. Nutrients 14, 64. doi: 10.3390/nu14010064. PMID: 35010939 PMC8746300

[B58] Koren O. Goodrich J. K. Cullender T. C. Spor A. Laitinen K. Bäckhed H. K. . (2012). Host remodeling of the gut microbiome and metabolic changes during pregnancy. Cell. 150, 470–480. doi: 10.1016/j.cell.2012.07.008. PMID: 22863002 PMC3505857

[B59] Kruskal W. H. Wallis W. A. (1952). Use of ranks in one-criterion variance analysis. J. Am. Stat. Assoc. 47, 583–621. doi: 10.1080/01621459.1952.10483441. PMID: 41909888

[B60] Kwon H. K. Seong J. K. (2021). New insights into the microbiota of wild mice. Mamm. Genome 32, 311–318. doi: 10.1007/s00335-021-09887-z. PMID: 34241667 PMC8295133

[B61] Legendre P. Legendre L. (2012). Numerical ecology (Amsterdam: Elsevier), 499.

[B62] Leite G. Pimentel M. Barlow G. M. Chang C. Hosseini A. Wang J. . (2021). “ Age and the aging process significantly alter the small bowel microbiome,” in Cell reports. (Cambridge MA: Cell Press) vol. 36. Available online at: https://www.cell.com/cell-reports/abstract/S2211-1247(21)01219-5. 10.1016/j.celrep.2021.109765 34592155

[B63] Li J. Ma Y. Bao Z. Gui X. Li A. N. Yang Z. . (2020). Clostridiales are predominant microbes that mediate psychiatric disorders. J. Psychiatr. Res. 130, 48–56. doi: 10.1016/j.jpsychires.2020.07.018. PMID: 32781373

[B64] Li X. Shi X. Yao Y. Shen Y. Wu X. Cai T. . (2023). Effects of stool sample preservation methods on gut microbiota biodiversity: new original data and systematic review with meta-analysis. Microbiol. Spectr. 11, e04297-22. doi: 10.2139/ssrn.4193760. PMID: 37093040 PMC10269478

[B65] Liang J. Q. Li T. Nakatsu G. Chen Y. X. Yau T. O. Chu E. . (2020). A novel faecal Lachnoclostridium marker for the non-invasive diagnosis of colorectal adenoma and cancer | Gut. Gut 69, 1248–1257. doi: 10.1136/gutjnl-2019-318532. PMID: 31776231 PMC7306980

[B66] Lin H. Eggesbø M. Peddada S. D. (2022). Linear and nonlinear correlation estimators unveil undescribed taxa interactions in microbiome data. Nat. Commun. 13, 4946. doi: 10.1038/s41467-022-32243-x. PMID: 35999204 PMC9399263

[B67] Lin H. Peddada S. D. (2020). Analysis of compositions of microbiomes with bias correction. Nat. Commun. 11, 3514. doi: 10.1038/s41467-020-17041-7. PMID: 32665548 PMC7360769

[B68] Lin H. Peddada S. D. (2024). Multigroup analysis of compositions of microbiomes with covariate adjustments and repeated measures. Nat. Methods 21, 83–91. doi: 10.1038/s41592-023-02092-7. PMID: 38158428 PMC10776411

[B69] Lozupone C. A. Hamady M. Kelley S. T. Knight R. (2007). Quantitative and qualitative β diversity measures lead to different insights into factors that structure microbial communities. Appl. Environ. Microbiol. 73, 1576–1585. doi: 10.1128/aem.01996-06. PMID: 17220268 PMC1828774

[B70] Lozupone C. Knight R. (2005). UniFrac: a new phylogenetic method for comparing microbial communities. Appl. Environ. Microbiol. 71, 8228–8235. doi: 10.1128/aem.71.12.8228-8235.2005. PMID: 16332807 PMC1317376

[B71] Lozupone C. Lladser M. E. Knights D. Stombaugh J. Knight R. (2011). UniFrac: an effective distance metric for microbial community comparison. ISME J. 5, 169–172. doi: 10.1038/ismej.2010.133. PMID: 20827291 PMC3105689

[B72] Lu J. Zhang S. Huang Y. Qian J. Tan B. Qian X. . (2022). Periodontitis-related salivary microbiota aggravates Alzheimer’s disease via gut-brain axis crosstalk. Gut Microbes 14, 2126272. doi: 10.1080/19490976.2022.2126272. PMID: 36175166 PMC9542625

[B73] Lubin J. B. Green J. Maddux S. Denu L. Duranova T. Lanza M. . (2023). Arresting microbiome development limits immune system maturation and resistance to infection in mice. Cell. Host Microbe 31, 554–570.e7. doi: 10.1016/j.chom.2023.03.006. PMID: 36996818 PMC10935632

[B74] Mandal R. K. Mandal A. Denny J. E. Namazii R. John C. C. Schmidt N. W. (2023). Gut Bacteroides act in a microbial consortium to cause susceptibility to severe malaria. Nat. Commun. 14, 6465. doi: 10.1038/s41467-023-42235-0. PMID: 37833304 PMC10575898

[B75] Maritan E. Quagliariello A. Frago E. Patarnello T. Martino M. E. (2024). The role of animal hosts in shaping gut microbiome variation. Philos. Trans. R. Soc Lond. B. Biol. Sci. 379, 20230071. doi: 10.1098/rstb.2023.0071. PMID: 38497257 PMC10945410

[B76] Markle J. G. M. Frank D. N. Mortin-Toth S. Robertson C. E. Feazel L. M. Rolle-Kampczyk U. . (2013). Sex differences in the gut microbiome drive hormone-dependent regulation of autoimmunity. Science 339, 1084–1088. doi: 10.1126/science.1233521. PMID: 23328391

[B77] Marotz C. Cavagnero K. J. Song S. J. McDonald D. Wandro S. Humphrey G. . (2021). Evaluation of the effect of storage methods on fecal, saliva, and skin microbiome composition. mSystems 6. doi: 10.1128/msystems.01329-20. PMID: 33906915 PMC8092129

[B78] Marquina D. Buczek M. Ronquist F. Łukasik P. (2021). The effect of ethanol concentration on the morphological and molecular preservation of insects for biodiversity studies. PeerJ 9, e10799. doi: 10.7717/peerj.10799. PMID: 33614282 PMC7883690

[B79] Martinson J. N. V. Pinkham N. V. Peters G. W. Cho H. Heng J. Rauch M. . (2019). Rethinking gut microbiome residency and the Enterobacteriaceae in healthy human adults. ISME J. 13, 2306–2318. doi: 10.1038/s41396-019-0435-7. PMID: 31089259 PMC6776003

[B80] Matějková T. Dodoková A. Kreisinger J. Stopka P. Stopková R. (2024). Microbial, proteomic, and metabolomic profiling of the estrous cycle in wild house mice. Microbiol. Spectr. 12, e02037-23. doi: 10.1128/spectrum.02037-23, PMID: 38171017 PMC10846187

[B81] Maurice C. F. CL Knowles S. Ladau J. Pollard K. S. Fenton A. Pedersen A. B. . (2015). Marked seasonal variation in the wild mouse gut microbiota. ISME J. 9, 2423–2434. doi: 10.1038/ismej.2015.53. PMID: 26023870 PMC4611506

[B82] McDonald D. Clemente J. C. Kuczynski J. Rideout J. R. Stombaugh J. Wendel D. . (2012). The Biological Observation Matrix (BIOM) format or: how I learned to stop worrying and love the ome-ome. GigaScience 1, 7. doi: 10.1186/2047-217x-1-7. PMID: 23587224 PMC3626512

[B83] McDonald D. Vázquez-Baeza Y. Koslicki D. McClelland J. Reeve N. Xu Z. . (2018). Striped UniFrac: enabling microbiome analysis at unprecedented scale. Nat. Methods 15, 847–848. doi: 10.1038/s41592-018-0187-8. PMID: 30377368 PMC7250580

[B84] McKinney W. (2010). “ Data structures for statistical computing in Python”, in: Proceedings of the 9th Python in Science Conference, 51–56.

[B85] Meng S. Chen B. Yang J. Wang J. Zhu D. Meng Q. . (2018). Study of microbiomes in aseptically collected samples of human breast tissue using needle biopsy and the potential role of in situ tissue microbiomes for promoting Malignancy. Front. Oncol. 8. doi: 10.3389/fonc.2018.00318, PMID: 30175072 PMC6107834

[B86] Michels N. Zouiouich S. Vanderbauwhede B. Vanacker J. Indave Ruiz B. I. Huybrechts I. (2022). Human microbiome and metabolic health: an overview of systematic reviews. Obes. Rev. 23, e13409. doi: 10.1111/obr.13409. PMID: 34978141

[B87] Mills J. N. Childs J. E. Ksiazek T. G. Peters C. J. Velleca W. M. (1995). Methods for trapping and sampling small mammals for virologic testing. Available online at: https://stacks.cdc.gov/view/cdc/11507.

[B88] Milovic A. Bassam K. Shao H. Chatzistamou I. Tufts D. M. Diuk-Wasser M. . (2020). Lactobacilli and other gastrointestinal microbiota of Peromyscus leucopus, reservoir host for agents of Lyme disease and other zoonoses in North America. PLoS One 15, e0231801. doi: 10.1371/journal.pone.0231801. PMID: 32817657 PMC7446861

[B89] Mistrick J. Kipp E. J. Weinberg S. I. Adams C. C. Larsen P. A. Craft M. E. (2024). Microbiome diversity and zoonotic bacterial pathogen prevalence in Peromyscus mice from agricultural landscapes and synanthropic habitat. Mol. Ecol. 33, e17309. doi: 10.1111/mec.17309. PMID: 38429967

[B90] Muegge B. D. Kuczynski J. Knights D. Clemente J. C. González A. Fontana L. . (2011). Diet drives convergence in gut microbiome functions across mammalian phylogeny and within humans. Science 332, 970–974. doi: 10.1126/science.1198719. PMID: 21596990 PMC3303602

[B91] Nobs S. P. Elinav E. (2019). Walk on the wildling side. Science 365, 444–445. doi: 10.1126/science.aay2864. PMID: 31371599

[B92] Odamaki T. Kato K. Sugahara H. Hashikura N. Takahashi S. Xiao J. Z. . (2016). Age-related changes in gut microbiota composition from newborn to centenarian: a cross-sectional study. BMC Microbiol. 16, 90. doi: 10.1186/s12866-016-0708-5. PMID: 27220822 PMC4879732

[B93] Oñate F. P. Chamignon C. Burz S. D. Lapaque N. Monnoye M. Philippe C. . (2023). Adlercreutzia equolifaciens is an anti-inflammatory commensal bacterium with decreased abundance in gut microbiota of patients with metabolic liver disease. Int. J. Mol. Sci. 24, 12232. doi: 10.3390/ijms241512232, PMID: 37569608 PMC10418321

[B94] Péré-Védrenne C. Flahou B. Loke M. F. Ménard A. Vadivelu J. (2017). Other Helicobacters, gastric and gut microbiota. Helicobacter 22, e12407. doi: 10.1111/hel.12407, PMID: 28891140

[B95] Qin Q. Yan S. Yang Y. Chen J. Li T. Gao X. . (2021). A metagenome-wide association study of the gut microbiome and metabolic syndrome. Front. Microbiol. 12. doi: 10.3389/fmicb.2021.682721, PMID: 34335505 PMC8322780

[B96] Reed A. W. Kennedy P. K. Beck M. L. Kennedy M. L. (2004). Using morphologic characters to identify Peromyscus in sympatry. Am. Midl. Nat. 152, 190–195. doi: 10.1674/0003-0031(2004)152[0190:umctip]2.0.co;2

[B97] Rosshart S. P. Herz J. Vassallo B. G. Hunter A. Wall M. K. Badger J. H. . (2019). Laboratory mice born to wild mice have natural microbiota and model human immune responses. Science 365, eaaw4361. doi: 10.1126/science.aaw4361. PMID: 31371577 PMC7377314

[B98] Ruiz-Saavedra S. Arboleya S. Nogacka A. M. González del Rey C. Suárez A. Diaz Y. . (2024). Commensal fecal microbiota profiles associated with initial stages of intestinal mucosa damage: a pilot study. Cancers 16, 104. doi: 10.3390/cancers16010104. PMID: 38201530 PMC10778549

[B99] Russell V. L. Stevens M. H. H. Zeisler A. A. Jezkova T. (2022). Identifying regional environmental factors driving differences in climatic niche overlap in Peromyscus mice. J. Mammal. 103, 45–56. doi: 10.1093/jmammal/gyab126. PMID: 40809543

[B100] Salazar-Jaramillo L. de la Cuesta-Zuluaga J. Chica L. A. Cadavid M. Ley R. E. Reyes A. . (2024). Gut microbiome diversity within Clostridia is negatively associated with human obesity. mSystems 9, e00627-24. doi: 10.1128/msystems.00627-24. PMID: 39012154 PMC11334427

[B101] Scheepers I. M. Cryan J. F. Bastiaanssen T. F. S. Rea K. Clarke G. Jaspan H. B. . (2020). Natural compulsive-like behaviour in the deer mouse (Peromyscus maniculatus bairdii) is associated with altered gut microbiota composition. Eur. J. Neurosci. 51, 1419–1427. doi: 10.1111/ejn.14610. PMID: 31663195

[B102] Schloissnig S. Arumugam M. Sunagawa S. Mitreva M. Tap J. Zhu A. . (2013). Genomic variation landscape of the human gut microbiome. Nature 493, 45–53. doi: 10.1038/nature11711. PMID: 23222524 PMC3536929

[B103] Schmidt E. Mykytczuk N. Schulte-Hostedde A. I. (2019). Effects of the captive and wild environment on diversity of the gut microbiome of deer mice (Peromyscus maniculatus). ISME J. 13, 1293–1305. doi: 10.1038/s41396-019-0345-8. PMID: 30664674 PMC6474230

[B104] Schriefer A. E. Cliften P. F. Hibberd M. C. Sawyer C. Brown-Kennerly V. Burcea L. . (2018). A multi-amplicon 16S rRNA sequencing and analysis method for improved taxonomic profiling of bacterial communities. J. Microbiol. Methods 154, 6–13. doi: 10.1016/j.mimet.2018.09.019. PMID: 30273610 PMC6375304

[B105] Shannon C. E. (1948). A mathematical theory of communication. Bell Syst. Tech. J. 27, 379–423, 623–656. doi: 10.1145/584091.584093. PMID: 41889584

[B106] Sikes R. S. Gannon W. L. the Animal Care and Use Committee of the American Society of Mammalogists (2011). Guidelines of the American Society of Mammalogists for the use of wild mammals in research. J. Mammal. 92, 235–253. doi: 10.1644/06-mamm-f-185r1.1. PMID: 29692469 PMC5909806

[B107] Sisk-Hackworth L. Brown J. Sau L. Levine A. A. Tam L. Y. I. Ramesh A. . (2023). Genetic hypogonadal mouse model reveals niche-specific influence of reproductive axis and sex on intestinal microbial communities. Biol. Sex Differ. 14, 79. doi: 10.1186/s13293-023-00564-1. PMID: 37932822 PMC10626657

[B108] Socała K. Doboszewska U. Szopa A. Serefko A. Włodarczyk M. Zielińska A. . (2021). The role of microbiota-gut-brain axis in neuropsychiatric and neurological disorders. Pharmacol. Res. 172, 105840. doi: 10.1016/j.phrs.2021.105840, PMID: 34450312

[B109] Stackebrandt E. Koch C. Gvozdiak O. Schumann P. (1995). Taxonomic dissection of the genus Micrococcus: Kocuria gen. nov. Nesterenkonia gen. nov. Kytococcus gen. nov. Dermacoccus gen. nov. and Micrococcus Cohn 1872 gen. emend. Int. J. Syst. Evol. Microbiol. 45, 682–699. doi: 10.1099/00207713-46-1-366. PMID: 7547287

[B110] Sweeny A. R. Lemon H. Ibrahim A. Watt K. A. Wilson K. Childs D. Z. . (2023). A mixed-model approach for estimating drivers of microbiota community composition and differential taxonomic abundance. mSystems. 8. doi: 10.1128/msystems.00040-23, PMID: 37489890 PMC10469806

[B111] Tabacaru C. A. Millar J. S. Longstaffe F. J. (2011). Seasonal moulting in deer mice (Peromyscys maniculatus) in the Rocky Mountains, Alberta. Can. Filed-Naturalist 125, 126–131. doi: 10.22621/cfn.v125i2.1195

[B112] Tang Q. Huang H. Xu H. Xia H. Zhang C. Ye D. . (2024). Endogenous Coriobacteriaceae enriched by a high-fat diet promotes colorectal tumorigenesis through the CPT1A-ERK axis. NPJ Biofilms Microbiomes 10, 1–15. doi: 10.1038/s41522-023-00472-7. PMID: 38245554 PMC10799938

[B113] Tavella T. Rampelli S. Guidarelli G. Bazzocchi A. Gasperini C. Pujos-Guillot E. . (2021). Elevated gut microbiome abundance of Christensenellaceae, Porphyromonadaceae and Rikenellaceae is associated with reduced visceral adipose tissue and healthier metabolic profile in Italian elderly. Gut Microbes 13, 1880221. doi: 10.1080/19490976.2021.1880221. PMID: 33557667 PMC7889099

[B114] Trigunaite A. Dimo J. Jørgensen T. N. (2015). Suppressive effects of androgens on the immune system. Cell. Immunol. 294, 87–94. doi: 10.1016/j.cellimm.2015.02.004. PMID: 25708485

[B115] Vacca M. Celano G. Calabrese F. M. Portincasa P. Gobbetti M. De Angelis M. (2020). The controversial role of human gut Lachnospiraceae. Microorganisms 8, 573. doi: 10.3390/microorganisms8040573. PMID: 32326636 PMC7232163

[B116] Vandeputte D. De Commer L. Tito R. Y. Kathagen G. Sabino J. Vermeire S. . (2021). Temporal variability in quantitative human gut microbiome profiles and implications for clinical research. Nat. Commun. 12, 6740. doi: 10.1038/s41467-021-27098-7. PMID: 34795283 PMC8602282

[B117] Vasar M. Davison J. Sepp S. K. Mucina L. Oja J. Al-Quraishy S. . (2022). Global soil microbiomes: a new frontline of biome-ecology research. Global Ecol. Biogeogr. 31, 1120–1132. doi: 10.1111/geb.13487. PMID: 41940437

[B118] Vázquez L. B. Cameron G. N. Medellín R. A. (2004). Characteristics of diet of Peromyscus aztecus and Reithrodontomys fulvescens in montane western Mexico. J. Mammal. 85, 196–205. doi: 10.1644/BRG-108

[B119] Vázquez-Baeza Y. Gonzalez A. Smarr L. McDonald D. Morton J. T. Navas-Molina J. A. . (2017). Bringing the dynamic microbiome to life with animations. Cell. Host Microbe 21, 7–10. doi: 10.1016/j.chom.2016.12.009. PMID: 28081445

[B120] Vázquez-Baeza Y. Pirrung M. Gonzalez A. Knight R. (2013). EMPeror: a tool for visualizing high-throughput microbial community data. GigaScience 2, 16. doi: 10.1186/2047-217x-2-16. PMID: 24280061 PMC4076506

[B121] Wald E. (2024). The Effects of Urbanization on Peromyscus sonoriensis: A Microbiome Analysis. Available online at: https://hdl.handle.net/20.500.12647/10907.

[B122] Wallace J. G. Potts R. H. Szamosi J. C. Surette M. G. Sloboda D. M. (2018). The murine female intestinal microbiota does not shift throughout the estrous cycle. PLoS One 13, e0200729. doi: 10.1371/journal.pone.0200729. PMID: 30011327 PMC6047814

[B123] Walter J. O’Toole P. W. (2023). Microbe profile: the lactobacillaceae. Microbiology 169, 1414. doi: 10.1099/mic.0.001414. PMID: 38088348 PMC10765037

[B124] Wang J. Linnenbrink M. Künzel S. Fernandes R. Nadeau M. J. Rosenstiel P. . (2014). Dietary history contributes to enterotype-like clustering and functional metagenomic content in the intestinal microbiome of wild mice. Proc. Natl. Acad. Sci. 111, E2703–E2710. doi: 10.1073/pnas.1402342111. PMID: 24912178 PMC4084472

[B125] Weiss S. Xu Z. Z. Peddada S. Amir A. Bittinger K. Gonzalez A. . (2017). Normalization and microbial differential abundance strategies depend upon data characteristics. Microbiome 5, 27. doi: 10.1186/s40168-017-0237-y. PMID: 28253908 PMC5335496

[B126] Weldon L. Abolins S. Lenzi L. Bourne C. Riley E. M. Viney M. (2015). The gut microbiota of wild mice. PLoS One 10, e0134643. doi: 10.1371/journal.pone.0134643. PMID: 26258484 PMC4530874

[B127] Whitmer S. L. M. Whitesell A. Mobley M. Talundzic E. Shedroff E. Cossaboom C. M. . (2024). “ Human orthohantavirus disease prevalence and genotype distribution in the U.S. 2008–2020: a retrospective observational study,” in The lancet regional health – americas. (Amsterdam: Elsevier) vol. 37. Available online at: https://www.thelancet.com/journals/lanam/article/PIIS2667-193X(24)00163-7/fulltext. 10.1016/j.lana.2024.100836 PMC11296052 39100240

[B128] Witmer G. W. Moulton R. S. (2012). “ Deer mice (Peromyscus spp.) biology, damage, and management: a review,” in Proceedings of the vertebrate pest conference, vol. 25. . Available online at: https://escholarship.org/uc/item/5p31k1gj.

[B129] Wolff J. O. Dueser R. D. Berry K. S. (1985). Food habits of sympatric Peromyscus leucopus and Peromyscus maniculatus. J. Mammal. 66, 795–798. doi: 10.2307/1380812. PMID: 39964225

[B130] Wolff J. O. Hurlbutt B. (1982). Day refuges of Peromyscus leucopus and Peromyscus maniculatus. J. Mammal. 63, 666–667. doi: 10.2307/1380276. PMID: 39964225

[B131] Wolmarans D. W. Scheepers I. M. Stein D. J. Harvey B. H. (2018). Peromyscus maniculatus bairdii as a naturalistic mammalian model of obsessive-compulsive disorder: current status and future challenges. Metab. Brain Dis. 33, 443–455. doi: 10.1007/s11011-017-0161-7. PMID: 29214602

[B132] Worsley S. F. Davies C. S. Lee C. Z. Mannarelli M. E. Burke T. Komdeur J. . (2024). Longitudinal gut microbiome dynamics in relation to age and senescence in a wild animal population. Mol. Ecol. 33, e17477. doi: 10.1111/mec.17477. PMID: 39010794

[B133] Wu C. S. Muthyala S. D. V. Klemashevich C. Ufondu A. U. Menon R. Chen Z. . (2021). Age-dependent remodeling of gut microbiome and host serum metabolome in mice. Aging (Albany NY) 13, 6330–6345. doi: 10.18632/aging.202525. PMID: 33612480 PMC7993679

[B134] Xiao Y. Shi Y. Ni Y. Ni M. Yang Y. Zhang X. (2024). Gestational diabetes-combined excess weight gain exacerbates gut microbiota dysbiosis in newborns, associated with reduced abundance of Clostridium, Coriobacteriaceae, and Collinsella. Front. Cell. Infect. Microbiol. 14. doi: 10.3389/fcimb.2024.1496447. PMID: 39726807 PMC11670820

[B135] Yang Z. Liu X. Wu Y. Peng J. Wei H. (2022). Effect of the microbiome on intestinal innate immune development in early life and the potential strategy of early intervention. Front. Immunol. 13. doi: 10.3389/fimmu.2022.936300, PMID: 35928828 PMC9344006

[B136] Yatsunenko T. Rey F. E. Manary M. J. Trehan I. Dominguez-Bello M. G. Contreras M. . (2012). Human gut microbiome viewed across age and geography. Nature 486, 222–227. doi: 10.1038/nature11053. PMID: 22699611 PMC3376388

[B137] Yilmaz E. Yalamanchali A. Dwidar M. E. Geiger J. L. Koyfman S. Chan T. A. . (2022). Lachnoclostridium and immune inflamed gene expression signature association in head & neck cancers. Int. J. Radiat. Oncol. Biol. Phys. 112, e37. doi: 10.1016/j.ijrobp.2021.12.086. PMID: 41940325

[B138] Yin H. Yang L. Peng G. Yang K. Mi Y. Hu X. . (2021). The commensal consortium of the gut microbiome is associated with favorable responses to anti-programmed death protein 1 (PD-1) therapy in thoracic neoplasms. Cancer Biol. Med. 18, 1040–1052. doi: 10.20892/j.issn.2095-3941.2020.0450. PMID: 33960176 PMC8610161

[B139] Yu Z. Wang Q. Wang Z. Liu S. Xia T. Duan C. . (2025). Lachnoclostridium intestinal flora is associated with immunotherapy efficacy in nasopharyngeal carcinoma. Head Neck 47, 269–281. doi: 10.1007/978-3-642-23499-6_96. PMID: 39135356

[B140] Zeng H. Ishaq S. L. Zhao F. Q. Wright A. D. G. (2016). Colonic inflammation accompanies an increase of β-catenin signaling and Lachnospiraceae/Streptococcaceae bacteria in the hind gut of high-fat diet-fed mice. J. Nutr. Biochem. 35, 30–36. doi: 10.1016/j.jnutbio.2016.05.015. PMID: 27362974

[B141] Zhang C. Chen F. Shen Y. Chen Y. Ma J. (2022). Sleep apnea is associated with the increase of certain genera of Ruminococcaceae and Lachnospiraceae in the gut microbiome of hypertensive patients. Expert Rev. Respir. Med. 16, 1247–1256. doi: 10.1080/17476348.2022.2147509. PMID: 36369876

[B142] Zhang J. Liu M. Ke S. Huang X. Fang S. He M. . (2021). Gut and vagina microbiota associated with estrus return of weaning sows and its correlation with the changes in serum metabolites. Front. Microbiol. 12. doi: 10.3389/fmicb.2021.690091, PMID: 34489885 PMC8417050

[B143] Zhang M. Zhang M. Kou G. Li Y. (2023). The relationship between gut microbiota and inflammatory response, learning and memory in mice by sleep deprivation. Front. Cell. Infect. Microbiol. 13. doi: 10.3389/fcimb.2023.1159771, PMID: 37293204 PMC10244646

[B144] Zheng L. Kelly C. J. Colgan S. P. (2015). Physiologic hypoxia and oxygen homeostasis in the healthy intestine. A review in the theme: cellular responses to hypoxia. Am. J. Physiol. Cell Physiol. 309, C350–C360. doi: 10.1152/ajpcell.00191.2015. PMID: 26179603 PMC4572369

