# Supplementary material for: Characterization of the gut microbiome of wild Peromyscus sonoriensis in New Mexico, USA
Source: Front Microbiomes. 2026 Apr 24;5:1672092. doi: 10.3389/frmbi.2026.1672092 (PMC13153134; doi:10.3389/frmbi.2026.1672092)
Supplement: Supplementary file 1 [file Image1.pdf]

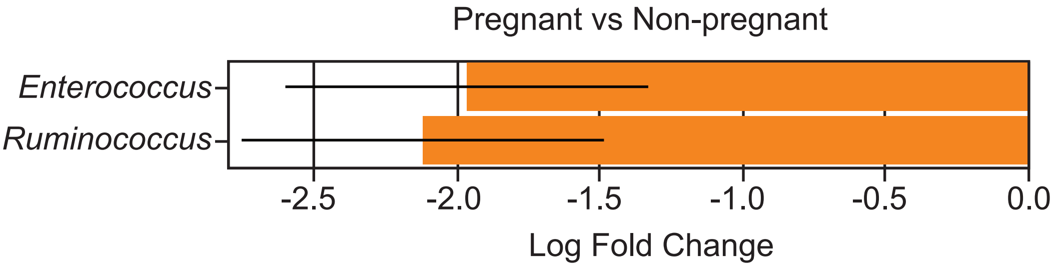

Supplemental figure S1. Differential bacterial family abundance in pregnant versus non-pregnant mice. Female mice from the Taos site were categorized based on their reproductive status at the time of capture. Pregnant animals had significant reductions in the genera *Enterococcus* and *Ruminococcus* compared to non-pregnant animals. Significance was calculated at the p = 0.05 level and was determined using ANCOMBC.
