# Supplementary material for: Characterization of the gut microbiome of wild Peromyscus sonoriensis in New Mexico, USA
Source: Front Microbiomes. 2026 Apr 24;5:1672092. doi: 10.3389/frmbi.2026.1672092 (PMC13153134; doi:10.3389/frmbi.2026.1672092)
Supplement: Supplementary file 2 [file Image2.pdf]

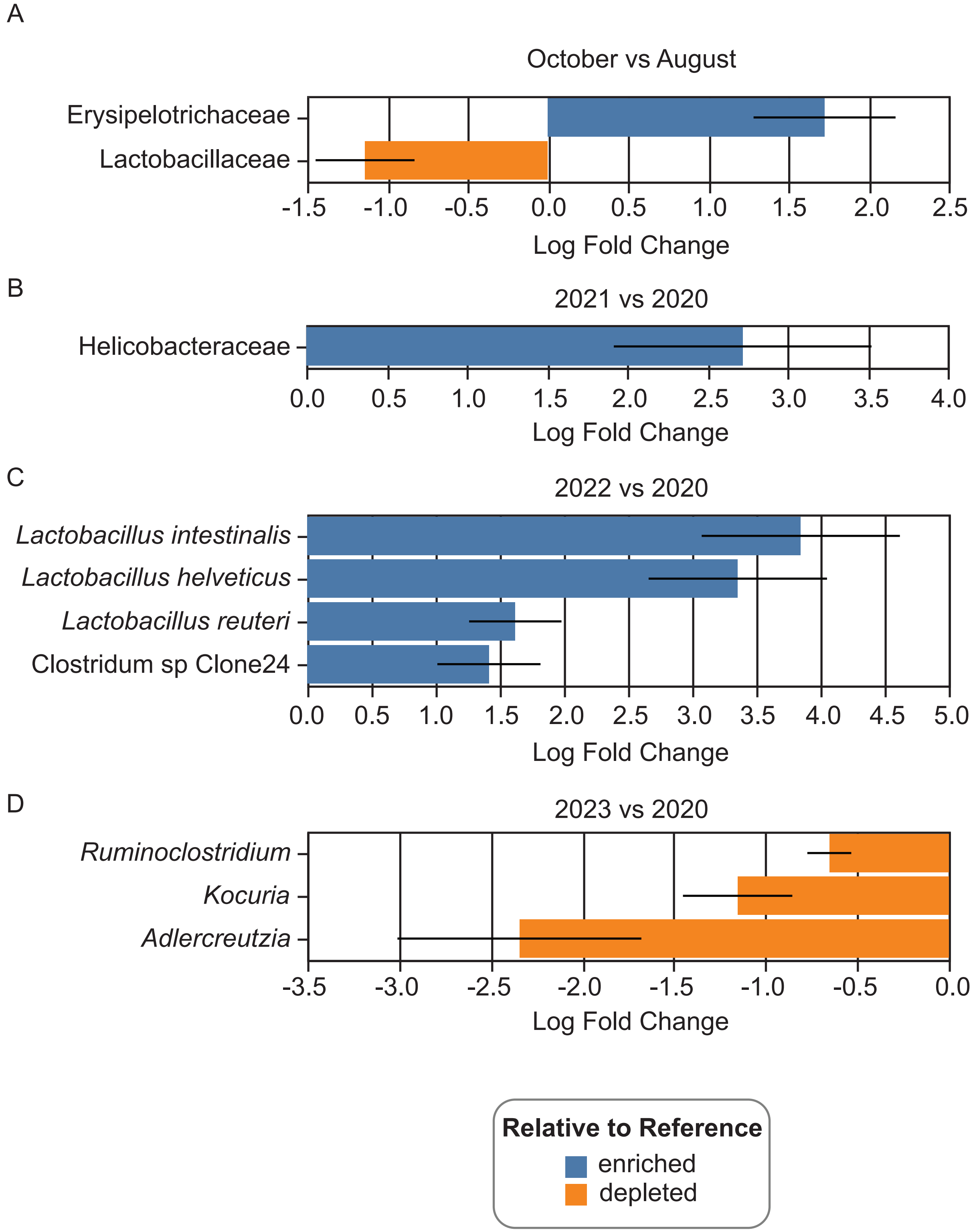

Supplemental figure S2. Differential bacterial abundance in relation to season and year of capture. A) Differentially present bacterial families at the McGaffey trapping location in October compared to August. There was an increase in family Erysipelotrichaceae and a decrease in family Lactobacillaceae. B) Differentially present bacterial families in year 2021 compared to 2020 at the Taos trapping site. There was an increase in family Helicobacteraceae. C) Differential bacterial species in year 2022 compared to 2020 at the Taos trapping site. There were increases in three *Lactobacillus* species: *intestinalis*, *helveticus*, and *reuteri*. There was an increase in one uncategorized species of clostridium. D) Differentially present bacterial genera in 2023 compared to 2020 at the Taos trapping site. There were reductions in *Ruminoclostridium*, *Kocuria*, and *Aldercreutzia*. All differential bacteria were determined at the family, genus, and species levels and the highest of the three levels with significance was reported. Significance was determined at the p = 0.05 level using ANCOMBC.
