# Supplementary material for: Characterization of the gut microbiome of wild Peromyscus sonoriensis in New Mexico, USA
Source: Front Microbiomes. 2026 Apr 24;5:1672092. doi: 10.3389/frmbi.2026.1672092 (PMC13153134; doi:10.3389/frmbi.2026.1672092)
Supplement: Supplementary file 3 [file Image3.pdf]

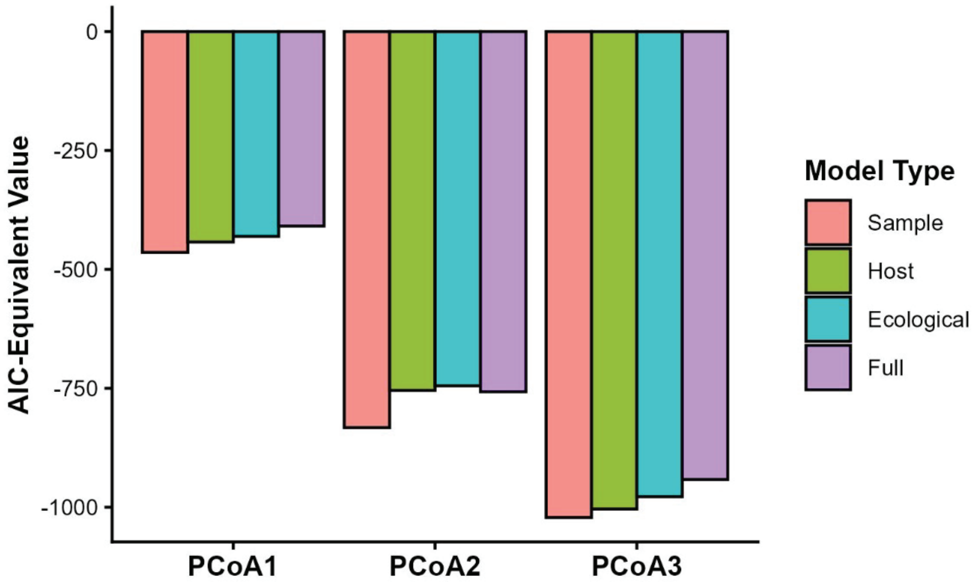

Supplemental figure S3: Performance of different GLMM models in explaining variation in beta diversity.
The full model includes all variables. The ecological model includes location. The host model includes age and sex. The sample model includes storage conditions. The full model performed the best, had the lowest AIC-equivalent value, for axes 1 and 3 in PCoA. The ecological model perfromed the best in axis 2.
