# Supplementary material for: Characterization of the gut microbiome of wild Peromyscus sonoriensis in New Mexico, USA
Source: Front Microbiomes. 2026 Apr 24;5:1672092. doi: 10.3389/frmbi.2026.1672092 (PMC13153134; doi:10.3389/frmbi.2026.1672092)
Supplement: Supplementary file 5 [file Table2.docx]

|  | PCoA1 | PCoA2 | PCoA3 |
| --- | --- | --- | --- |
| model_sample | -464.2670 | -832.7031 | -1021.6941 |
| model_host | -442.3265 | -754.2827 | -1003.6286 |
| model_eco | -430.6286 | -744.8749 | -977.8882 |
| model_full | **-408.7612** | **-757.3371** | **-941.5787** |

Supplemental table 2: performance of different GLMM models at explaining variation in beta diversity. Values are AIC-equivalent values. Lower values indicate improved performance.
