# Supplementary material for: Characterization of the gut microbiome of wild Peromyscus sonoriensis in New Mexico, USA
Source: Front Microbiomes. 2026 Apr 24;5:1672092. doi: 10.3389/frmbi.2026.1672092 (PMC13153134; doi:10.3389/frmbi.2026.1672092)
Supplement: Supplementary file 6 [file Table3.docx]

|  | signif_taxa |
| --- | --- |
| model_sample | 6 |
| model_host | 6 |
| model_eco | 7 |
| model_full | 7 |

Supplemental table 3: total differentially abundant bacterial families based on ANCOMBC analysis with GLMM.
